# Privacy-preserving parallel kNN classification algorithm using index-based filtering in cloud computing

**DOI:** 10.1371/journal.pone.0267908

**Published:** 2022-05-05

**Authors:** Yong-Ki Kim, Hyeong-Jin Kim, Hyunjo Lee, Jae-Woo Chang

**Affiliations:** 1 Dept. of IT Convergence System, Vision College of Jeonju, Jeonju, Jeollabuk-do, Republic of Korea; 2 Dept. of Computer Engineering, Chonbuk National University, Jeonju, Jeollabuk-do, Republic of Korea; Victoria University, AUSTRALIA

## Abstract

With the development of cloud computing, interest in database outsourcing has recently increased. In cloud computing, it is necessary to protect the sensitive information of data owners and authorized users. For this, data mining techniques over encrypted data have been studied to protect the original database, user queries and data access patterns. The typical data mining technique is kNN classification which is widely used for data analysis and artificial intelligence. However, existing works do not provide a sufficient level of efficiency for a large amount of encrypted data. To solve this problem, in this paper, we propose a privacy-preserving parallel kNN classification algorithm. To reduce the computation cost for encryption, we propose an improved secure protocol by using an encrypted random value pool. To reduce the query processing time, we not only design a parallel algorithm, but also adopt a garbled circuit. In addition, the security analysis of the proposed algorithm is performed to prove its data protection, query protection, and access pattern protection. Through our performance evaluation, the proposed algorithm shows about 2∼25 times better performance compared with existing algorithms.

## 1 Introduction

With the growing popularity of cloud computing, there has been growing interest in outsourcing databases. Cloud computing provides a service that allows internet-connected users to use virtual computing resources such as storage, computation, and network. Thus, a cloud service provider can maintain computing resources rapidly and flexibly. A data owner can reduce efforts to purchase, install, and expand computing systems, and mitigate the constraints of physical space. Cloud computing is attracting a lot of attention from individuals and companies because it can reduce the cost of system maintenance and data management, and can utilize computing resources needed without expertise. Meanwhile, we should consider three requirements in an outsourced database. First, it is necessary to protect the database because the database contains sensitive information of the data owner [1, 2]. Second, the query and the query result should not be exposed because personal information related to user preference may be uncovered. Third, data access patterns should be protected because the cloud provider is able to infer private information from the data access pattern.

**Fig 1:**
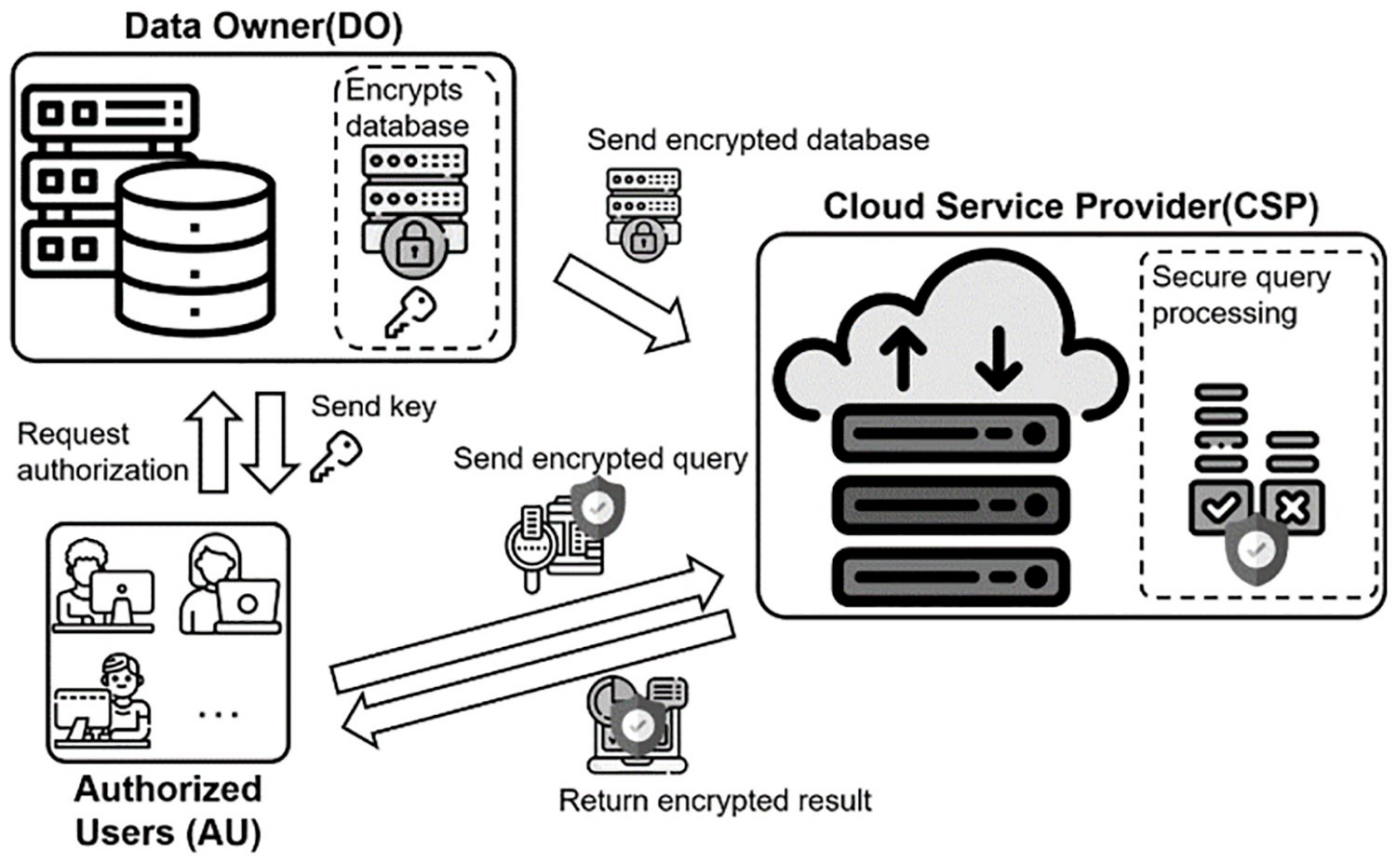
Database outsourcing model.

Therefore, Data Mining over Encrypted Data (DMED) has been studied to protect the original database, user queries and data access patterns. Early studies modify plaintexts to substituted data and outsources them to a cloud [3–7]. However, these early studies have a disadvantage in that they cannot completely protect data and queries because they are vulnerable to various attacks such as chosen-plaintext attacks. To solve this problem, recent studies encrypt the database and outsource the encrypted database to the cloud [8–15]. Before a data owner outsources his/her database to a cloud service provider (cloud provider), he/she encrypts the database. The cloud provider processes the query received from an authorized user. The cloud provider can perform data management and system maintenance instead of the data owner. The authorized user can directly request the desired results from the cloud provider. The process of query processing over the outsourced database is shown in Fig 1.

Among DMED, the kNN classification algorithm is widely used for three reasons. First, the kNN classification algorithm has a relatively higher accuracy than other classification algorithms. Second, with the addition of more data, the kNN classification algorithm constantly evolves and is capable of quickly adapting to the changes in input dataset. Finally, the kNN classification algorithm gives a user a flexibility to choose a distance measure metric. Therefore, the kNN classification algorithm is used for various applications such as pattern analysis, image analysis, and user analysis [16].

Samanthula et al.’s work [16] and Kim et al.’s work [17] proposed kNN classification algorithms based on homomorphic encryption which can support various operations without decryption. Recent studies can also support data privacy, query privacy and hiding data access patterns. However, while processing the kNN classification algorithm, the recent works require high computation cost because they need to add random noise data to prevent exposure of the original data. Moreover, they require a large amount of processing time for kNN classification over the encrypted database. To the best of our knowledge, there is no existing parallel kNN classification algorithm which is suitable for processing a large amount of encrypted data.

The motivation of this paper is as follows. First, the existing algorithms suffer from high computational cost by using encrypted binary array to perform comparison operations. Therefore, we aim at reducing computational cost by proposing secure comparison protocol based on Yao’s garbled circuit. Second, the existing algorithms require high data encryption cost. To deal with this problem, we propose an improved secure protocol by using an encrypted random value pool. Finally, to the best of our knowledge, there is no existing parallel kNN classification algorithm. We aim at designing a parallel kNN classification algorithm for processing a large amount of encrypted data.

The contributions of this paper are as follows.

- Supporting privacy preservation: By processing queries using homomorphic encryption without data decryption, we can protect the confidentiality of both data and user’s queries while hiding data access patterns from an attacker.
- Reducing computation cost: By using the improved secure protocol based on an encrypted random value pool, we can reduce the high computation cost of the random value generation for the data encryption.
- Improving the performance of kNN classification: By proposing a new parallel kNN classification algorithm, we can reduce the amount of processing time for kNN classification.

The rest of this paper is as follows. In Section 2, we introduce the existing works on kNN classification algorithms over the encrypted database. In Section 3, we describe the overall system architecture and propose secure protocols for the proposed parallel kNN classification algorithm. In Section 4, we propose a parallel kNN classification algorithm that preserves both data and query privacy on the cloud. In Section 5, we provide the security proof of our kNN classification algorithm. In Section 6, we perform a performance analysis of the proposed algorithm. In Section 7, we describe the impact of the proposed parallel classification algorithm as a discussion. Finally, in Section 8, we conclude our paper with the future work.

## 2 Background and related work

### 2.1) Background

#### 2.1.1 Paillier cryptosystem

The Paillier cryptosystem is a probabilistic asymmetric algorithm for public key cryptography [18]. In the Paillier cryptosystem, the encryption key pk is given as (N, g), where N is the multiplication value between two large prime numbers p and q in *Z*_*N*^2^_. Here, g is a random integer value at *Z*_*N*^2^_ where *Z*_*N*^2^_ denotes an integer domain ranged from 0 to *Z*_*N*^2^_. Meanwhile, the decryption key sk is given as (p, q). The Paillier cryptosystem has the following characteristics. First the Paillier cryptosystem can support homomorphic addition and multiplication. Assume that the encryption function of the Paillier cryptosystem is E(.) and its decryption function is D(.), For two encrypted data E(a) and E(b), the product E(a) × E(b) is equal to E(a+b), which is the encrypted value of the plaintext a+b, as shown in Eq (1).

$$\begin{array}{c} E\left(a+b\right)=E\left(a\right)\times E\left(b\right)\;mod\;n^{2} \end{array}$$ (1)

For two plaintexts a and b, the *b*^*t*^*h* power of the encrypted data E(a), i.e, *E*(*a*)^*b*^, is equal to E(a × b), which is the encrypted value of the plaintext a × b, as shown in Eq (2).

$$\begin{array}{c} E\left(a\times b\right)=E{\left(a\right)}^{b}\;mod\;n^{2} \end{array}$$ (2)

Second, the Paillier cryptosystem supports semantic security where only negligible information about the plaintext can be feasibly extracted from the ciphertext. Specifically, any probabilistic, polynomial-time algorithm (PPTA), which is given the ciphertext of a certain message m and its length, cannot determine any partial information on the message with a probability higher than all other PPTA’s that only have access to the message length [19]. This concept is the computational complexity similar to Shannon’s concept of perfect secrecy. Perfect secrecy means that the ciphertext reveals no information at all about the plaintext, whereas semantic security implies that any information revealed cannot be feasibly extracted.

#### 2.1.2 Attack model

In the outsourcing database environment, two attack models can be considered: a semi-honest attack model and a malicious attack model [20]. In the semi-honest (or honest-but-curious) attack model, the cloud performs its own protocol honestly, but attempts to obtain sensitive data about the data owner and the authorized user during the protocol execution. To prevent a semi-honest attack, sensitive data must always be protected. A malicious attack model attempts to acquire sensitive data by deviating from a given secure protocol. Because a secure protocol can be contaminated by a malicious attack, it is difficult to recover the secure protocol. To protect sensitive data against the malicious attack model, a defender focuses on detecting attacks and recovering the damaged secure protocol. Since we aim at protecting sensitive data in cloud computing, we design our algorithm based on the semi-honest attack model. A secure protocol for the semi-honest attack model is defined as follows [17].

**Definition 1**. Assuming that *a*_*i*_ is the input data of cloud *C*_*i*_, ∏_*i*_(*π*) is the execution image of *C*_*i*_ for the protocol *π* and *b*_*i*_ is the result data of *C*_*i*_ executing the *π* protocol. If the execution image ∏_*Si*_(*π*) simulating *π* is computationally indistinguishable from ∏_*i*_(*π*), the protocol *π* is said to be a secure protocol for the semi-honest attack model.

In Definition 1, the execution image generally includes the input data and output data of the protocol. The security of the protocol under the semi-honest attack model can be verified by showing that the protocol’s execution image does not expose the cloud’s data.

### 2.2) Related work

#### 2.2.1 B. Yao et al.’s work

B. Yao et al. proposed a secure kNN classification algorithm [21] based on a partition-based secure Voronoi diagram (SVD) [22]. The SVD relies on any standard encryption scheme E such as public-key encryption RSA and symmetric-key encryption AES, rather than using any new encryption schemes. Because the SVD is as secure as E for any standard security model in which E is proven secure, the SVD is indistinguishable in either chosen plaintext or chosen ciphertext attacks. To process the secure kNN classification queries, the algorithm retrieves the relevant encrypted partition instead of finding the encrypted exact k-nearest neighbors. However, most of the computations are performed locally by the end-user while processing the kNN classification query. As a result, the algorithm conflicts the purpose of outsourcing the DBMS functionalities to the cloud. Furthermore, the algorithm leaks data access patterns to the cloud, such as the partition ID corresponding to a user query.

#### 2.2.2 B. K. Samanthual et al.’s work

B. K. Samanthula et. al. proposed a secure k-NN classification algorithm, denoted by PPkNN, over encrypted data in the cloud [16]. PPkNN can protect the confidentiality of the data, user’s input query, and data access patterns. PPkNN mainly consists of two stages: the secure retrieval of k-nearest neighbors and the secure computation of majority class. In the secure retrieval of k-nearest neighbors, a query user initially sends his query q (in encrypted form) to *C*_1_. Then, *C*_1_ and *C*_2_ involve in a set of sub-protocols to securely retrieve the class labels corresponding to the k-nearest neighbors of the input query q. At the end of this step, the encrypted class labels of the k-nearest neighbors are known only to *C*_1_. In the secure computation of the majority class, *C*_1_ and *C*_2_ jointly compute the class label with majority voting among the k-nearest neighbors of q. At the end of this step, only the query user knows the class label corresponding to input query record q. However, PPkNN requires a very high computation cost for hiding data access patterns.

#### 2.2.3 H. Kim et al.’s work

H. Kim et. al. proposed a secure kNN classification algorithm which uses both the Paillier cryptosystem and an encrypted kd-tree index [17]. The Paillier cryptosystem is a homomorphic encryption scheme which is indistinguishable in either chosen-plaintext or chosen-ciphertext attacks, so that the cloud can process the kNN classification queries without decrypting any data or a user’s query. Before outsourcing data to the cloud, a data owner builds a kd-tree index and encrypts both the original database and the leaf nodes of the kd-tree index. Therefore, the algorithm can protect the data, the query and the data access pattern. By using the encrypted kd-tree index, the algorithm can reduce the amount of query processing time. However, because the algorithm must generate encrypted random values for privacy-preserving, it requires a high computation cost.

#### 2.2.4 W. Wu et al.’s work

W. Wu et al. proposed a privacy preserving kNN classification algorithm over encrypted database in outsourced cloud environments [23]. The algorithm newly generates unique classification label keys for each user through a secure three-party protocol. The keys are used to re-encrypt the labels into new ciphertexts that can only be decrypted by the corresponding user. The algorithm hides the data access patterns from a federated cloud server which performs the process of kNN classification by using two non-colluding clouds. However, the algorithm conflicts the purpose of outsourcing the DBMS functionalities to the cloud because both the data owner and authorized users must participate in the process of label re-encryption.

#### 2.2.5 Y. Tan et al.’s work

Y. Tan et al. proposed a lightweight edge-based privacy-preserving kNN classification algorithm over a hybrid encrypted cloud database [24]. A data owner can upload his/her database to the cloud server, and an authorized user can send a query to the cloud server to execute kNN queries. The algorithm is performed against the semi-honest attack model. After the query is sent, the authorized user does not need to participate in the kNN classification. They also proposed a secure distance protocol in which the cloud servers cannot derive any private information from the authorized user. Compared with the SIP protocol in the state-of-the-art PPKC algorithm [16], the proposed secure distance protocol has less corrupted computation.

#### 2.2.6 J. Du and F. Bian’s work

J. Du and F. Bian proposed a non-interactive and efficient privacy-preserving kNN classification algorithm [25]. The algorithm is performed against the semi-honest attack model. To achieve privacy preservation, the algorithm encrypts all outsourced data and users’ query records by using two encryption schemes: order preserving encryption [26] and the Paillier cryptosystem [16]. To hide the data access pattern, the information in the cloud server is always maintained in ciphertext format. In terms of classification accuracy, the algorithm is proven to be very close to one using both plaintext data and the non-interactive encrypted data query scheme.

Table 1 shows the comparison of the existing studies. We explain their comparison with respect to three major factors. First, B. K. Samanthula et al.’s work [16], H. Kim et al.’s work [17], W. Wu et al.’s work [23] and Y. Tan et al.’s work [24] support hiding access pattern, while B. Yao et al.’s work [21] and J. Du and F. Bian’s work [25] do not support it. Second, W. Wu et al.’s work and Y. Tan et al.’s work require low computation overhead while B. K. Samanthula et al.’s work and H. Kim et al.’s work need high computation overhead. Finally, B. Yao et al.’s work, B. K. Samanthula et al.’s work, H. Kim et al.’s work and W. Wu et al.’s work have low risk in terms of security, while Y. Tan et al.’s work and J. Du and F. Bian’s work have high risk in terms of security.

**Table 1:** Comparison of existing studies.

	Hiding access patterns	Index	Computation overhead	Encryption	User involvement	Exact match / Approximate match	Security risk
B. Yao et al.’s work [21]	Not support	Secure Voronoi diagram	Moderate	Any Standard Encryption	Involved	Approximate match	Low
B.K. Samanthula et al.’s work [16]	Support	None	Very high	Paillier	Not involved	Exact match	Low
H. Kim et al.’s work [17]	Support	Encrypted kd-tree	High	Paillier	Not involved	Exact match	Low
W. Wu et al.’s work [23]	Support	None	Low	self-production	Involved	Exact match	Low
Y. Tan et al.’s work [24]	Support	None	Low	Paillier (data), ElGamal (Classification label)	Not involved	Exact match	High
J. Du and F. Bian’s work [25]	Not support	Encrypted kd-tree	Moderate	Paillier (label), Order preserving encryption(OPE)	Involved	Approximate match	High

## 3 Overall system architecture and secure protocols

### 3.1) System architecture

In the outsourcing database environment, two attack models can be considered: a malicious attack model and a semi- honest attack model [20]. In a malicious attack model, the cloud can deviate from the protocol procedure. A protocol against malicious attack model is inefficient because it requires exceedingly high cost. In the semi-honest attack model, the cloud correctly follows the given protocol, but tries to acquire the sensitive information of both the data owner and the query issuer. However, a protocol against a semi-honest attack model is practical because the cloud has a higher level of authority than outsider attackers. Therefore, according to earlier work [16, 17], we also adopt the semi-honest attack model. Table 2 shows a list of notations used in this paper. Our system architecture supports secure protocols between clouds by performing Secure Multiparty Computation (SMC). SMC is based on multi-party data processing in which several entities cooperate to perform calculations for deriving specific results. For this, the following factors must be satisfied to achieve the result of secure protocols while avoiding data leakage.

**Table 2:** Definitions of common notations.

Notations	Description
*E*(⋅), *D*(⋅)	Encryption function and decryption function
*t*_*i*_, *t*_*i*,*j*_	*i*^*th*^ record and *j*^*th*^ attribute value of *i*^*th*^ record
$t_{i}^{\prime },t_{i,j}^{\prime }$	*i*^*th*^ extracted record during the index search and its *j*^*th*^ attribute value
*q*, *q*_*j*_	A query of a user and *j*^*th*^ attribute value of a query q
*n*, *m*	The total number of data and attributes in T
*cnt*	The number of data extracted during the index search step
*h*	Level of the kd-tree
*node*	*z*^*th*^ node of the kd-tree
*node*_*z*_.*t*_*s*,*j*_	*j*^*th*^ attribute of *s*^*th*^ record stored in *z*^*th*^ node of the kd-tree
*lb*_*z*,*j*_, *ub*_*z*,*j*_	*j*^*th*^ attribute value of lower/upper bound of *z*^*th*^ kd-tree node
*F*	Fan-out(maximum # of data in each leaf node)
*α*_*z*_	Output of SCMP or SPE protocol for node
*α*	A set of values consisting of *α*
*l*	Domain size (in bits)
*r*	Random integers
*L*	Array for entire label
*w*	The number of labels
*L*′	k-array of labels for k-nearest neighbors

#### 3.1.1 Input privacy

No information about private data held by multiple parties can be inferred from the messages sent during the protocol execution. The only information that can be inferred about private data is whatever could be inferred from seeing the output of the function alone.

#### 3.1.2 Correctness

Any proper subset of adversarial colluding parties that is willing to share information or deviate from the instructions during the protocol execution should not be able to force honest parties to output an incorrect result. This correctness goal comes in two categories: either the honest parties are guaranteed to compute the correct output (a robust SMC protocol), or the honest parties abort if they find an error (an SMC protocol with abort).

**Fig 2:**
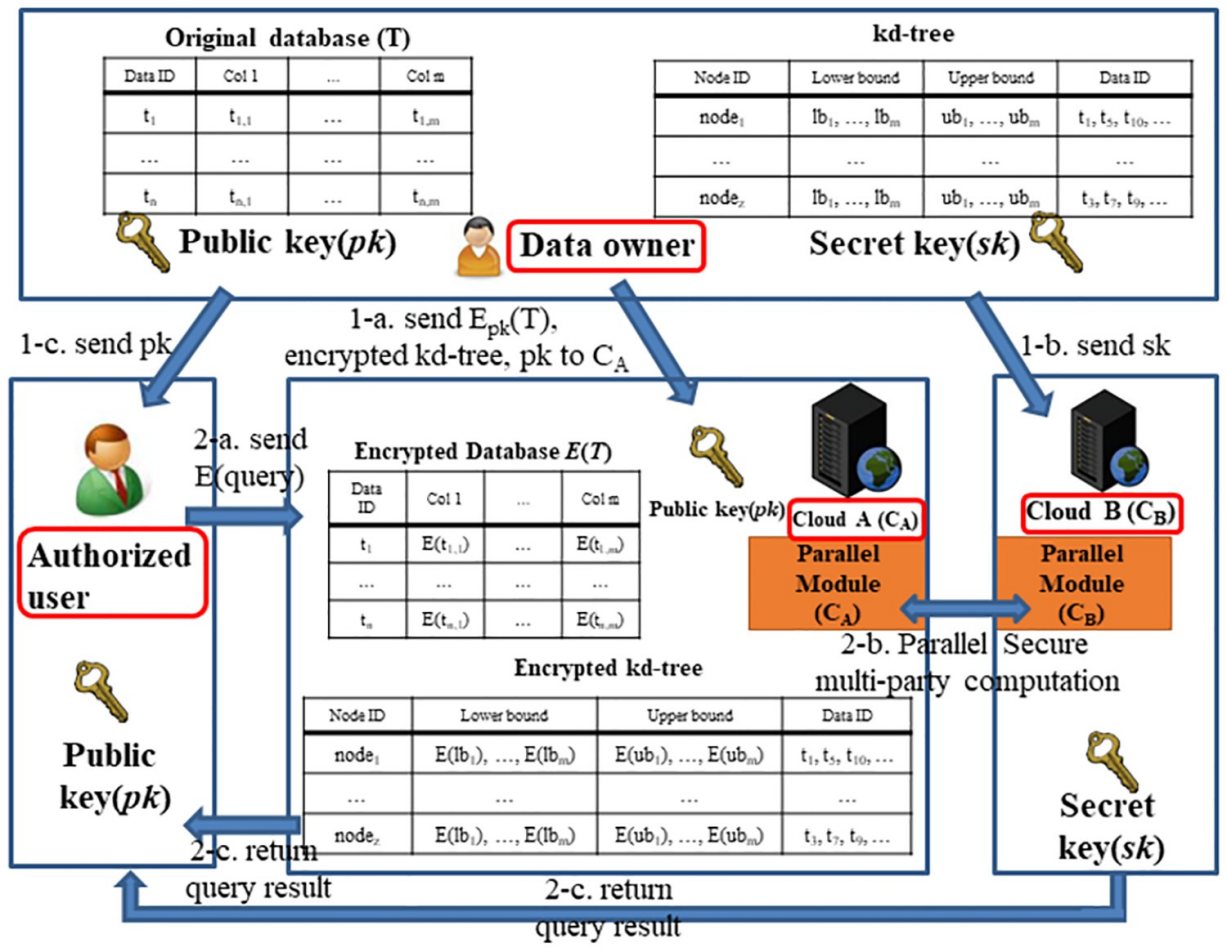
Overall system architecture.

Fig 2 shows the overall system architecture. The data owner holds the original database T consisting of n records *t*_*i*_ (1 ≤ *i* ≤ *n*). Each record *t*_*i*_ includes m attributes (or columns) and one label. Here, we call the *j*^*th*^ attribute of the *i*^*th*^ record as *t*_*i*,*j*_(1 ≤ *i* ≤ *n*, 1 ≤ *j* ≤ *m* + 1). First, the data owner partitions the original data by using the kd-tree index. Assuming that the level of the constructed kd-tree is h, the total number of leaf nodes is 2^*h*−1^. In the leaf node, an attribute stores its region information, i.e., a lower bound *lb*_*z*,*j*_ and an upper bound *ub*_*z*,*j*_, where 1 ≤ *z* ≤ 2^*h*−1^ and 1 ≤ *j* ≤ *m*. Second, the data owner generates an encryption public key (*pk*) and a decryption secret key (*sk*) based on the Paillier cryptosystem [18]. Third, the data owner encrypts the database with the Paillier cryptosystem to protect the original data. Because the unit of the encryption is the attribute of each record, *E*(*t*_*i*,*j*_) (1 ≤ *i* ≤ *n*, 1 ≤ *j* ≤ *m* + 1) is generated. Finally, the leaf node of the constructed kd-tree is encrypted because the data owner needs to protect the data access pattern. Because the unit of the encryption is the attributes of each leaf node, *E*(*lb*_*z*,*j*_) and *E*(*ub*_*z*,*j*_) are generated(1 ≤ *z* ≤ 2^*h*−1^, 1 ≤ *j* ≤ *m*).

### 3.2) Secure protocols

#### 3.2.1 Encrypted random value pool

To support data privacy in a cloud computing environment, the existing works [16, 17] prevent *C*_*B*_ from extracting meaningful information (Fig 2) while executing a secure protocol by using the Paillier cryptosystem. However, they require high computation cost because the secure protocol generates an encrypted random value for protecting the original data. Therefore, we propose an encrypted random value pool to reduce the computation cost for encryption. Before *C*_*A*_ processes a query (Fig 2), we generate the random plaintext from *Z*_*N*_ and store the encrypted random plaintext into an encrypted random value pool. While processing a query in *C*_*A*_, a random ciphertext is selected from the encrypted random value pool whenever a secure protocol is called. Therefore, while processing a secure protocol, *C*_*A*_ not only prevents *C*_*B*_ from extracting meaningful information, but also reduces the cost of generating encrypted random values. Table 3 shows a comparison of the number of data encryptions for each secure protocol in our work and existing works [16, 17]. The Secure Multiplication protocol of the existing works requires three times as the number of encryptions as our work. The Secure Compare protocol used in B. K. Samanthula et al.’s work requires log_2_ *D* times as the number of encryptions as our work while the one used in H. Kim et al.’s work requires three times as the number of encryptions as our work.

**Table 3:** A comparison of amount in secure protocols.

algorithms	Secure Multiplication Protocol	Secure Compare Protocol
B. K. Samanthula et al.’s work	3 × *E*	*log*_2_ *D* × *E*
H. Kim et al’s work	3 × *E*	3 × *E*
Proposed algorithm	1 × *E*	1 × *E*

#### 3.2.2 Secure multiplication protocol using an encrypted random value pool

We propose a Secure Multiplication protocol using an Encrypted random value pool (SME protocol) which multiplies two encrypted values *E*(*α*) and *E*(*β*). Algorithm 1 shows the SME protocol. First, when two encrypted values *E*(*α*) and *E*(*β*) are given as inputs, *C*_*A*_ selects two random values *E*(*r*_*a*_) and *E*(*r*_*b*_) from the encrypted random value pool (line 1). Second, *C*_*A*_ calculates *E*(*α* + *r*_*a*_) and *E*(*β* + *r*_*b*_) by using Eq (1), then sends them to *C*_*B*_ (line 2∼3). Third, *C*_*B*_ decrypts *E*(*α* + *r*_*a*_) and *E*(*β* + *r*_*b*_) by using the secret key and calculates the multiplication of the two plaintext *α* + *r*_*a*_ and *β* + *r*_*b*_ (line 4). Fourth, *C*_*B*_ encrypts (*α* + *r*_*a*_) × (*β* + *r*_*b*_) and send it to *C*_*A*_ (line 5). Finally, *C*_*A*_ obtains *E*(*α* × *β*) by removing *α* × *r*_*b*_, *β* × *r*_*a*_ and *r*_*a*_ × *r*_*b*_ from the received value, where ‘*N*^−*x*^’ in the *Z*_*N*_ domain is the same as ‘-x’ (line 6).

**Algorithm 1** SME Protocol

**Input**: *E*(*α*), *E*(*β*)

**Output**: *E*(*α* × *β*)

*C*_*A*_:

1: Pick random value *E*(*r*_*a*_) and *E*(*r*_*b*_) in the encrypted random value pool

2: *E*(*α*′)←*E*(*α*) × *E*(*r*_*a*_);*E*(*β*′)←*E*(*β*) × *E*(*r*_*b*_)

3: Send *E*(*α*′), *E*(*β*′) to *C*_*B*_

*C*_*B*_:

4: *h* ← *D*(*E*(*α*′) × *D*(*E*(*β*′) mod *N* // *h* = *α* × *β* + *α* × *r*_*b*_ + *β* × *r*_*a*_ + *r*_*a*_ × *r*_*b*_

5: Send *E*(*h*) to *C*_*A*_

*C*_*A*_:

6: $E\left(\alpha \times \beta \right)\leftarrow E\left(h\right)\times E{\left(\alpha \right)}^{N-r_{b}}\times E{\left(\beta \right)}^{N-r_{a}}\times E{\left(r_{a}\times r_{b}\right)}^{N-1}$

#### 3.2.3 Garbled secure compare protocol using encrypted random value pool

We propose the Garbled Secure Compare protocol using an Encrypted random value pool (GSCE protocol) which is performed by using a garbled circuit consisting of two ADD gates and one CMP gate [27]. Assume that *E*(*u*) and *E*(*v*) are ciphertext for two plaintext *u* and *v*. When *E*(*u*) and *E*(*v*) are given to *C*_*A*_, the GSCE protocol returns *E*(1) if *u* ≤ *v* is satisfied, otherwise it returns *E*(0). Algorithm 2 shows the GSCE protocol. First, *C*_*A*_ selects two random value *E*(*r*_*u*_) and *E*(*r*_*v*_) from the encrypted random value pool (line 1). Second, *C*_*A*_ calculates *E*(*m*_1_) = *E*(*u*)^2^ × *E*(*r*_*u*_) and *E*(*m*_2_) = *E*(*v*)^2^ × *E*(1) × *E*(*r*_*v*_) (line 1∼2). Third, *C*_*A*_ randomly selects one of two random functions, i.e., *F*_0_ and *F*_1_. The selected random function is not disclosed to *C*_*B*_. If *C*_*A*_ selects *F*_0_, *C*_*A*_ sends an encrypted ordered pair <*E*(*m*_2_), *E*(*m*_1_)> to *C*_*B*_. If *C*_*A*_ selects *F*_1_, *C*_*A*_ sends an encrypted ordered pair <*E*(*m*_1_), *E*(*m*_2_)> to *C*_*B*_ (line 3∼7). Fourth, *C*_*B*_ decrypts the data received from *C*_*A*_ (line 8∼11). When *C*_*A*_ selects *F*_0_, *C*_*B*_ acquires an ordered pair <*m*_2_, *m*_1_>, otherwise *C*_*B*_ acquires an ordered pair <*m*_1_, *m*_2_>. Fifth, *C*_*A*_ creates a garbled circuit consisting of two ADD gates and one CMP gate. If *F*_0_ is selected, −*r*_*v*_ and −*r*_*u*_ are transferred to the first ADD gate and the second ADD gate, respectively. Otherwise, −*r*_*u*_ and −*r*_*v*_ are transferred to the first and the second ADD gates, respectively (lines 12∼16). Sixth, *C*_*B*_ transfers the first data to the first ADD gate, and the second data to the second ADD gate. Therefore, when *F*_0_ is selected, *C*_*B*_ transfers *m*_2_ and *m*_1_ to the first and the second ADD gates, respectively. Otherwise, *m*_1_ and *m*_2_ are transferred to the first and the second ADD gates, respectively (line 17∼20). Seventh, the first ADD gate adds two input values: −*r*_*v*_ and *m*_2_ for *F*_0_ and −*r*_*u*_ and m1 for *F*_1_. The result of the first ADD gate (*result*_1_) is transferred to the CMP gate (line 21∼24). Eighth, the second ADD gate adds two input values: −*r*_*u*_ and *m*_1_ for *F*_0_ and −*r*_*v*_ and *m*_2_ for *F*_1_. The result of the second ADD gate (*result*_2_) is transferred to the CMP gate (line 25∼28). Due to the characteristics of the garbled circuit, the exposure of any information does not occur in the ADD gate. Ninth, the CMP gate returns *α* = 1 if *result*_1_ ≤ *result*_2_, and *α* = 0 otherwise (line 29 30). Finally, the result *α* can be checked on *C*_*B*_ side, and *C*_*B*_ transmits *E*(*α*) to *C*_*A*_ (line 31). Because *C*_*B*_ does not know whether *F*_0_ or *F*_1_ is selected by *C*_*A*_, *C*_*B*_ cannot determine the result of comparison of *E*(*u*) and *E*(*v*). When *F*_0_ is selected, *C*_*A*_ changes *E*(*α*) through the SBN protocol [11] and returns *E*(*α*) (line 32∼34). Here, *C*_*A*_ cannot obtain the actual value of *α* due to the characteristics of the Pallier cryptosystem.

**Algorithm 2** GSCE Protocol

**Input**: *E*(*u*), *E*(*v*)

**Output**: *E*(1) when *u* ≤ *v*, *E*(0) otherwise

*C*_*A*_:

01: Pick random value *E*(*r*_*u*_) and *E*(*r*_*v*_) in the encrypted random value pool

02: *E*(*m*_1_)←*E*(*u*)^2^ × *E*(*r*_*u*_)

03: *E*(*m*_2_)←*E*(*v*)^2^ × *E*(1) × *E*(*r*_*v*_)

04: *h* ← *D*(*E*(*α*′) × *D*(*E*(*β*′) mod *N* // *h* = *α* × *β* + *α* × *r*_*b*_ + *β* × *r*_*a*_ + *r*_*a*_ × *r*_*b*_

05: Randomly choose *F*_0_ or *F*_1_

06: If *F*_0_ u>v is chosen, then

07:  Send <*E*(*m*_2_), *E*(*m*_1_)> to *C*_*B*_

08: else

09:  Send <*E*(*m*_1_), *E*(*m*_2_)> to *C*_*B*_

*C*_*B*_:

10: If *F*_0_ u>v is chosen, then

11:  Obtain <*m*_2_, *m*_1_> by decrypting <*E*(*m*_2_), *E*(*m*_1_)>

12: else

13:  Obtain <*m*_1_, *m*_2_> by decrypting <*E*(*m*_1_), *E*(*m*_2_)>

*C*_*A*_:

14: Generate garbled circuit

15: If *F*_0_ u>v is chosen, then

16:  Put −*r*_*v*_ and −*r*_*u*_ into 1^*st*^ and 2^*nd*^ ADD gates

17: else

18:  Put −*r*_*u*_ and −*r*_*v*_ into 1^*st*^ and 2^*nd*^ ADD gates

*C*_*B*_:

19: If *F*_0_ u>v is chosen, then

20:  Put *m*_2_ and *m*_1_ into 1^*st*^ and 2^*nd*^ ADD gates

21: else

22:  Put *m*_1_ and *m*_2_ into 1^*st*^ and 2^*nd*^ ADD gates

1^st^ ADD Gate:

24: If *F*_0_ u>v is chosen, then

25:  *result*_1_ = *calculate* − *r*_*v*_ + (*v* + *r*_*v*_)

26: else

27:  *result*_1_ = *calculate* − *r*_*u*_ + (*u* + *r*_*u*_)

2^*nd*^ ADD Gate:

29: If *F*_0_ u>v is chosen, then

30:  *result*_2_ = *calculate* − *r*_*u*_ + (*u* + *r*_*u*_)

31: else

32:  *result*_2_ = *calculate* − *r*_*v*_ + (*v* + *r*_*v*_)

CMP Gate

34: If *result*_1_ > *result*_2_ is chosen, then

35:  output *α* = 1 to *C*_*B*_

36: else

37:  output *α* = 0 to *C*_*B*_

*C*_*B*_:

38: *E*(*α*)← encrypt *α*

*C*_*A*_:

39: If *F*_0_ u>v is chosen, then

40:  *E*(*α*)←*SBN*(*E*(*α*))

41: Return *E*(*α*)

## 4 Privacy-preserving parallel kNN classification algorithm using index filtering

**Fig 3:**
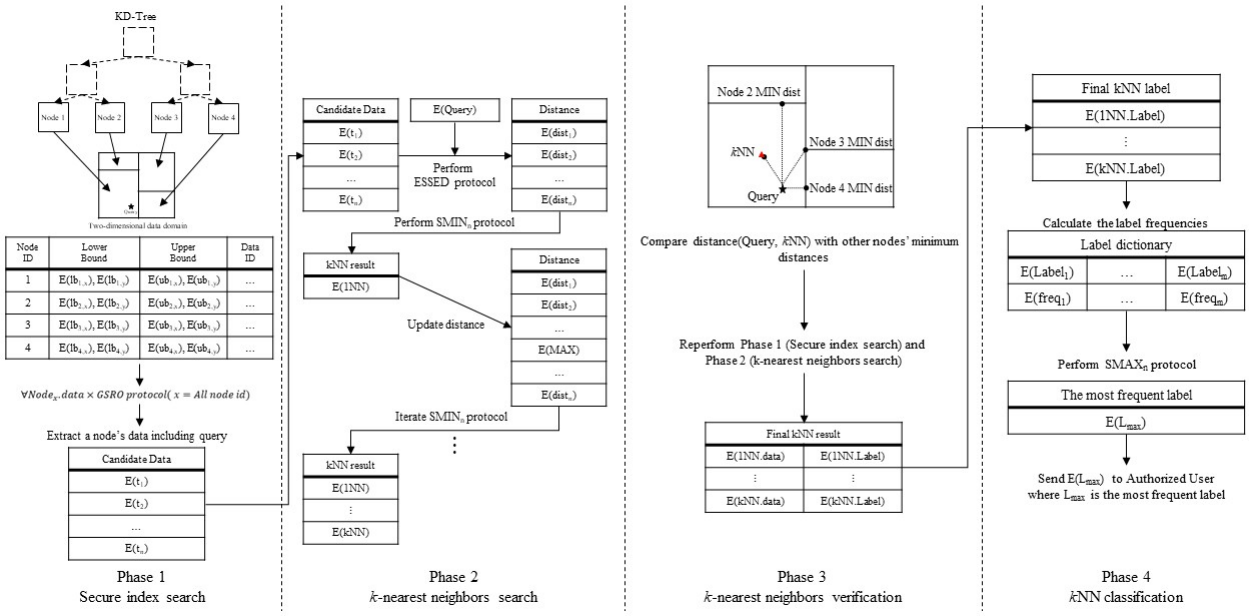
Proposed parallel kNN classification algorithm.

The proposed parallel kNN classification algorithm can support the protection of data, query, and data access pattern in a cloud computing environment. For this, the proposed privacy-preserving parallel kNN classification algorithm is composed of four phases: secure index search, k-nearest neighbors search, kNN verification, and kNN classification, as shown in Fig 3.

### 4.1) Secure index search phase

In the secure index search phase, the proposed algorithm determines the leaf node which includes the given query in the encrypted kd-tree. The procedure of the secure index search is shown in Algorithm 3. First, *C*_*A*_ makes *t* number of partitions and allocates them to the given threads (line 1). Here, *t* is calculated by dividing the number of leaf nodes by the number of threads. Second, by using the GSRO protocol, the algorithm finds which leaf node includes the query in each thread. If a node includes the query, the GSRO protocol returns *E*(1), otherwise the protocol returns *E*(0). The result of the GSRO protocol is stored in an array *E*(*α*). The algorithm randomly reorders the members of the array *E*(*α*) and transfers the reordered array *E*(*α*′) to *C*_*B*_(line 2∼7). Third, *C*_*B*_ decrypts the array *E*(*α*′) and makes groups by allocating the decrypted members uniformly based on the number of 1s. If a node has the decrypted value of 1, it becomes a seed of a group. *C*_*B*_ sends groups to *C*_*A*_ (line 8–15). Finally, *C*_*A*_ extracts all the encrypted data in the node corresponding to *E*(1). If a node has *E*(1), the algorithm can safely extract the data of the node because the node includes the query. Otherwise, the algorithm can remove the data of the node because it does not include the query (line 16∼30).

**Algorithm 3** Secure Index Search

**Input**: $kd-tree=E\left(node_{1}\right),...,E\left(node_{Num_{Node}}\right),query=E\left(q\right)$

**Output**: $E\left(\alpha_{1}\right),...,E\left(\alpha_{Num_{Node}}\right)|\alpha \in \left(0,1\right)$

*C*_*A*_:

01: t = *NumNode*/*NumThread*

02: Run thread

03: for 1 ≤ *i* ≤ *NumThread*

04:  for *t* × (*i* − 1)≤*j* ≤ *t* × *i*

05:   *E*(*α*_*j*_) = *GSRO*(*E*(*q*), *E*(*q*), *E*(*range*_*j*_.*lb*), *E*(*range*_*j*_.*ub*))

06: Terminate thread

07: *E*(*α*′) = ∏(*E*(*α*)); Send *E*(*α*′) to *C*_*B*_

*C*_*B*_:

08: *α*′ = *D*(*E*(*α*′))

09: c = the number of’1’ in *α*′

10: Create c number of node groups

11: for each node group

12:  assign a node with *α*′ = 1

13:  assign (*num*_*node*_/*c*) − 1 nodes with *α*′ = 0

14:  shuffle the sequence of nodes

15: Send node group to *C*_*A*_

*C*_*A*_:

16: cnt = 0

17: for each node group

18:  permute node IDs using *Π*^−1^

19: t = F/NumThread

20: Run thread

21: for 1 ≤ *i* ≤ *NumThread*

22:  for each node group

23:   for *t* × (*i* − 1)≤*s* ≤ *t* × *i*

24:    for 1 ≤ *z* ≤ *num* //num is # of nodes in the selected group

25:     $E\left(t_{z,j}^{\prime }\right)\leftarrow SM\left(node_{z}.t_{s,j},E\left(\alpha_{z}\right)\right)$ for 1 ≤ *j* ≤ *m* + 1

26:    for 1 ≤ *j* ≤ *m* + 1

27:     $E\left(cand_{cnt,j}\right)\leftarrow \prod_{z=1}^{num}E\left(t_{z,j}^{\prime }\right)$

28:    *cnt* ← *cnt* + 1

29: Terminate thread

30: return E(cand)

### 4.2) k-Nearest neighbors search phase

In the k-nearest neighbors search phase, our algorithm finds k-nearest points among the encrypted candidates which are extracted from the index search phase. The procedure of the k-nearest neighbors search is shown in Algorithm 4. First, *C*_*A*_ calculates the squared Euclidean distance set *E*(*d*_*i*_) (1 ≤ *i* ≤ *cnt*, where *cnt* is the number of candidates) between the query and the encrypted candidates through the ESSED protocol [17] in a parallel way (line 1∼6). Second, *C*_*A*_ finds the minimum value *E*(*d*_*min*_) among *E*(*d*_*i*_)(1 ≤ *i* ≤ *cnt*) through the *SMS*_*n*_ protocol [16] (line 8–10). Additionally, *C*_*A*_ calculates the difference between *E*(*d*_*min*_) and *E*(*d*_*i*_) (1 ≤ *i* ≤ *cnt*) by using *E*(*d*_*min*_) × *E*(*d*_*i*_)^*N*−1^, and stores the results into an array *E*(*τ*_*i*_) (1 ≤ *i* ≤ *cnt*). *C*_*A*_ makes $E(\tau_{i}^{\prime }$) (1 ≤ *i* ≤ *cnt*) by raising *E*(*τ*_*i*_) to the power of a random integer. *C*_*A*_ makes *E*(*β*^*i*^) (1 ≤ *i* ≤ *cnt*) by applying a shuffling function *π* to $E(\tau_{i}^{\prime })$ (1 ≤ *i* ≤ *cnt*) and sends it to *C*_*B*_ (line 11∼18). Therefore, the original distance and data access patterns are protected from *C*_*B*_. Third, if the *i*^*th*^ decrypted value of *E*(*β*_*i*_) (1 ≤ *i* ≤ *cnt*) is 0, *C*_*B*_ sets to *E*(1) the *i*^*th*^ value of a temporary array *E*(*U*_*i*_) (1 ≤ *i* ≤ *cnt*). Otherwise, *C*_*B*_ sets to *E*(0) the *i*^*th*^ value of a temporary array *E*(*U*_*i*_)(1 ≤ *i* ≤ *cnt*). *C*_*B*_ sends *E*(*U*_*i*_)(1 ≤ *i* ≤ *cnt*) to *C*_*A*_ (line 19∼22). Fourth, *C*_*A*_ makes *E*(*V*_*i*_)(1 ≤ *i* ≤ *cnt*) by applying a deshuffling function *π* − 1 to *E*(*U*_*i*_)(1 ≤ *i* ≤ *cnt*). *C*_*A*_ performs the SM protocol between *E*(*V*_*i*_)(1 ≤ *i* ≤ *cnt*) and *E*(*cand*_*i*,*j*_)(1 ≤ *i* ≤ *cnt* and 1 ≤ *j* ≤ *m* + 1, where *m* is the data dimension). *C*_*A*_ stores the result of the SM protocol in a temporary array $E(V_{i,j}^{\prime }$)(1 ≤ *i* ≤ *cnt* and 1 ≤ *j* ≤ *m* + 1). Next, *C*_*A*_ calculates Eq 3 by using Eq 1 (line 23∼31). Fifth, if the algorithm does not find k-nearest neighbors, *C*_*A*_ updates *E*(*d*_*i*_) (1 ≤ *i* ≤ *cnt*) by calculating Eq 4 in a parallel way, where *E*(*max*) is the maximum value of the data domain (line 32∼38). If *E*(*V*_*i*_) equals to *E*(1), *E*(*d*_*i*_) corresponding to *E*(*V*_*i*_) is updated to *E*(*max*) through Eq 4. Otherwise, *E*(*d*_*i*_) corresponding to *E*(*V*_*i*_) is maintained. Finally, *C*_*A*_ terminates the k-nearest neighbors search phase if k-nearest neighbors are found (line 39).

$$\begin{array}{c} E({t^{\prime }}_{s,j})=\prod_{i=1}^{cnt}E(V_{i,j})(1\leq j\leq m+1) \end{array}$$ (3)

$$\begin{array}{c} E\left(d_{i}\right)=SM(E\left(V_{i}\right),\;E\left(max\right)\times SM\left(SBN\left(E\left(V_{i}\right)\right),\;E\left(d_{i}\right)\right) \end{array}$$ (4)

**Algorithm 4** k-nearest neighbor search phase

**Input**: *E*(*q*), *E*(*cand*), *k*

**Output**: *t*′//*candidatekNNresult*

*C*_*A*_:

01: Run thread

02:  t = *NumNode*/*NumThread*

03:  for 1 ≤ *i* ≤ *NumThread*

04:   for *t* × (*i* − 1)≤*j* ≤ *t* × *i*

05:    *E*(*d*_*j*_) = *ESSED*(*E*(*q*), *E*(*cand*_*j*_))

06: Terminate thread

07: for 1 ≤ *s* ≤ *k*

08:  Run thread

09:   *E*(*d*_*min*_) = *SMS*_*n*_(*E*(*d*_1_), …, *E*(*d*_*cnt*_))

10:  Terminate thread

11:  Run thread

12:   t = cnt/NumThread

13:   for 1 ≤ *i* ≤ *NumThread*

14:    for *t* × (*i* − 1)≤*j* ≤ *t* × *i*

15:     *E*(*τ*_*j*_) = *E*(*d*_*min*_) × *E*(*d*_*j*_)^*N*−1^

16:     $E\left(\tau_{j}^{\prime }\right)=E{\left(\tau_{j}\right)}^{r_{i}}$

17:  Terminate thread

18:  *E*(*β*)←∏(*τ*_*j*_); Send *E*(*β*) to *C*_*B*_

*C*_*B*_:

19:  for 1 ≤ *i* ≤ *cnt*

20:   If *D*(*E*(*β*_*j*_)) = 0, then *E*(*U*_*i*_)←*E*(1)

21:   Else *E*(*U*_*i*_)←*E*(0)

22:  Send E(U) to *C*_*A*_

*C*_*A*_:

23:  *E*(*V*)←∏^−1^(*U*)

24:  Run thread

25:   *t* = *cnt*/*NumThread*

26:   for 1 ≤ *u* ≤ *NumThread*

27:    for *t* × (*u* − 1)≤*i* ≤ *t* × *u*

28:     for 1 ≤ *j* ≤ *m* + 1

29:      $E\left(V_{i,j}^{\prime }\right)\leftarrow SM\left(E\left(V_{i},E\left(cand_{i,j}\right)\right)\right)$

30:      $E\left(t_{s,j}^{\prime }\right)\leftarrow \prod_{i=1}^{cnt}E\left(V_{i,j}^{\prime }\right)$

31:  Terminate thread

32:  Run thread

33:   t = cnt/NumThread

34:   for 1 ≤ *i* ≤ *NumThread*

35:    for *t* × (*i* − 1)≤*j* ≤ *t* × *i*

36:     If *s* < *k* then,

37:      *E*(*d*_*j*_) = *SM*(*E*(*V*_*j*_), *E*(*max*)) × *SM*(*SBN*(*E*(*V*_*j*_)), *E*(*d*_*j*_))

38:  Terminate thread

39: return *E*(*t*′)

### 4.3) k-Nearest neighbors verification phase

In the k-nearest neighbors verification phase, the algorithm verifies whether the distance between the a node and the query(*E*(*q*) = <*E*(*q*_1_), *E*(*q*_2_), …, *E*(*q*_*m*_)>, where *m* is the data dimension) is shorter than the distance, *E*(*dist*_*k*_), between the query and *k*^*th*^ nearest neighbor ($E\left(t^{\prime }\right)=<E\left(t_{k,1}^{\prime }\right),E\left(t_{k,2}^{\prime }\right),\ldots ,E\left(t_{k,m}^{\prime }\right)>$). The procedure of the k-nearest neighbors verification phase is shown in Algorithm 5. First, *C*_*A*_ calculates *E*(*dist*_*k*_) between *E*(*q*) and $E(t_{k}^{\prime })$ using the ESSED protocol (line 1). Second, the algorithm performs the GSCE protocol between *E*(*q*_*j*_) and the lower bound of *node*_*z*_(*E*(*node*_*z*_.*lb*_*j*_) (1 ≤ *z* ≤ *num*_*node*_) for each dimension *j*(1 ≤ *j* ≤ *m*), and stores the result of the GSCE protocol into *E*(*ψ*_1,*j*_). If *E*(*q*_*j*_) (1 ≤ *j* ≤ *m*) is less than or equal to *E*(*node*_*z*_.*lb*_*j*_), *E*(*ψ*_1,*j*_) is *E*(1). Then, the algorithm performs the GSCE protocol between *E*(*q*_*j*_) (1 ≤ *j* ≤ *m*) and the upper bound of *node*_*z*_ (*E*(*node*_*z*_.*ub*_*j*_)(1 ≤ *z* ≤ *num*_*node*_) for each dimension *j*, and stores the result of the GSCE protocol into *E*(*ψ*_2,*j*_) (line 2∼5). If *E*(*q*_*j*_) is less than or equal to *E*(*node*_*z*_.*ub*_*j*_), *E*(*ψ*_2,*j*_) is *E*(1). Third, the algorithm performs the SBXOR protocol [16] between *E*(*ψ*_1,*j*_) and *E*(*ψ*_2,*j*_), and stores the result of the SBXOR protocol into *E*(*ψ*_3,*j*_) (line 6). Fourth, the algorithm calculates the shortest point of *node*_*z*_ (1 ≤ *z* ≤ *num*_*node*_), *E*(*sp*_*z*_) = < *E*(*sp*_*z*,1_), *E*(*sp*_*z*,2_), …, *E*(*sp*_*z*,*m*_) > where *m* is the data dimension, by using Eqs 5 and 6 (line 7∼10).

$$\begin{array}{c} f(E({lb}_{j}),E({ub}_{j}))=\;SM\left(E\left(\psi_{1,j}\right),E\left({lb}_{j}\right)\right)\times SM\left(SBN\left(\psi_{1,j}\right),E\left({ub}_{j}\right)\right) \end{array}$$ (5)

$$\begin{array}{c} E({sp}_{z,j})=SM(E(\psi_{3,j}),E(q_{j}))\times SM\left(SBN\left(E(\psi_{3,j})\right),\;f(E({lb}_{j}),\;E({ub}_{j}))\right) \end{array}$$ (6)

Fifth, *C*_*A*_ calculates the squared Euclidean distance between *E*(*q*) and *E*(*sp*_*z*_)(1 ≤ *z* ≤ *num*_*node*_) through the ESSED protocol and stores the result into the shortest distance of the *node*_*z*_, *E*(*spdist*_*z*_)(1 ≤ *z* ≤ *num*_*node*_) (line 11). In addition, *C*_*A*_ updates *E*(*spdist*_*z*_)(1 ≤ *z* ≤ *num*_*node*_) by using Eq 7 (line 12 13). *E*(*α*_*z*_) in Eq 7 is the result of the GSRO protocol in algorithm 1. This update avoids an unnecessary index search phase by updating the shortest distance of the node already searched in the previous phase.

$$\begin{array}{c} E({spdist}_{z})\leftarrow SM(E(\alpha_{z}),E(max))\times SM\left(SBN\left(E(\alpha_{z})\right),E({spdist}_{z})\right) \end{array}$$ (7)

Sixth, *C*_*A*_ performs the GSCE protocol between *E*(*spdist*_*z*_) and *E*(*dist*_*k*_), and stores the result into *E*(*α*_*z*_) (line 14). If *E*(*spdist*_*z*_) is less than *E*(*dist*_*k*_), the *node*_*z*_ needs additional searching. Finally, by performing lines 9∼33 of the secure index search phase, *C*_*A*_ extracts the encrypted data belonging to the *node*_*z*_ and adds them to *E*(*t*′). In addition, *C*_*A*_ obtains the kNN result array, *E*(*result*_*i*_)(1 ≤ *i* ≤ *k*), by performing the k-nearest neighbors search phase (line 15∼17). *C*_*A*_ stores the label of the k-nearest neighbors into $E\left(L_{i}^{\prime }\right)\left(1\leq i\leq k\right)$ (line 18∼19).

**Algorithm 5** k-nearest neighbors verification phase

**Input**: *E*(*q*), *E*(*node*), *E*(*t*′), *k*

**Output**: *result*

*C*_*A*_:

01: $E\left(dist_{k}\right)=ESSED\left(E\left(q\right),E\left(t_{k}^{\prime }\right)\right)$

02: for 1 ≤ *z* ≤ *num*_*node*_

03:  for 1 ≤ *j* ≤ *m*

04:   *E*(*ψ*_1_) = *GSCMP*(*E*(*q*_*j*_), *E*(*node*_*z*_.*lb*_*j*_))

05:   *E*(*ψ*_2_) = *GSCMP*(*E*(*q*_*j*_), *E*(*node*_*z*_.*ub*_*j*_))

06:   *E*(*ψ*_1_) = *SBXOR*(*E*(*ψ*_1_), *E*(*ψ*_2_))

07:   *E*(*temp*) = *SM*(*E*(*ψ*_1_), *E*(*node*_*z*_.*lb*_*j*_))

08:   *E*(*temp*)←*E*(*temp*) × *SM*(*SBN*(*E*(*ψ*_1_)), *E*(*node*_*z*_.*ub*_*j*_))

09:   *E*(*temp*) = *SM*(*E*(*temp*), *SBN*(*E*(*ψ*_3_)))

10:   *E*(*sp*_*z*,*j*_) = *E*(*temp*) × *SM*(*E*(*ψ*_1_), *E*(*q*_*j*_))

11:  *E*(*spdist*_*z*_) = *ESSED*(*E*(*q*), *E*(*sp*_*z*_))

12:  *E*(*temp*) = *SM*(*E*(*α*_*z*_), *E*(*max*))

13:  *E*(*spdist*_*z*_) = *E*(*temp*) × *SM*(*SBN*(*E*(*α*_*z*_)), *E*(*spdist*_*z*_))

14:  *E*(*α*_*z*_)←*GSCMP*(*E*(*spdist*_*z*_), *E*(*dist*_*k*_))

15: *E*(*t*″)← perform 7 ∼ 36 lines of Algorithm 1

16: *E*(*t*′)← append *E*(*t*″) to *E*(*t*′)

17: *result* ← *performAlgorithm*2

18: for 1 ≤ *i* ≤ *k*

19:  $E\left(L_{i}^{\prime }\right)=E\left(result_{i,m+1}\right)$

### 4.4) k-Nearest neighbors classification phase

In the kNN classification phase, the algorithm extracts the most frequent label from the label of the k-nearest neighbors, $E\left(L_{i}^{\prime }\right)\left(1\leq i\leq k\right)$. The procedure of the kNN classification phase is shown in Algorithm 6. *C*_*A*_ and *C*_*B*_ calculate the frequency of $E\left(L_{i}^{\prime }\right)\left(\;1\leq i\leq k\right)$ by using the secure frequency protocol [17] (line 1). The label with the highest frequency is selected (line 2). *C*_*A*_ adds a random integer *r*_*q*_ to the selected label and stores the result into a temporary variable *E*(*r*_*q*_) (line 3). *C*_*A*_ sends *E*(*r*_*q*_) to *C*_*B*_ and *r*_*q*_ to *AU* (line 4). *C*_*B*_ decrypts *E*(*r*_*q*_) and sends it to *AU* (line 5–6). *AU* obtains the final result by combining the results of *C*_*A*_ and *C*_*B*_ (line 7∼8).

**Algorithm 6** Knn classification

**Input**: $<E\left(L_{1}\right),...,E\left(L_{w}\right)>,<E\left(L_{1}^{\prime }\right),...,E\left(L_{w}^{\prime }\right)>$

**Output**: *E*(*L*_*q*_)

*C*_*A*_ and *C*_*B*_:

01: <*E*(*f*(*L*_1_), …, *E*(*f*(*L*_*w*_)))> = *SF*(Δ, Δ′), where $\Delta =<E\left(L_{1}\right),...,E\left(L_{w}\right)>,\Delta^{\prime }=<E\left(L_{1}^{\prime }\right),...,E\left(L_{w}^{\prime }\right)>$

02: (*f*(*max*), *E*(*L*_*q*_)) = *SXS*_*w*_(<*E*(*f*(*L*_1_)), …, *E*(*f*(*L*_*w*_)) >, < *E*(*L*_1_), …, *E*(*L*_*w*_)>)

*C*_*A*_:

03: *E*(λ_*q*_) = *E*(*c*_*q*_) × *E*(*r*_*q*_), where *r*_*q*_ ∈ *Z*_*N*_

04: Send *E*(λ_*q*_) to *C*_*B*_ and *r*_*q*_ to *AU*

*C*_*B*_:

05: Receive *E*(λ_*q*_) from *C*_*A*_

06: $\lambda_{q}^{\prime }=D\left(\lambda_{q}\right);$ Send $\lambda_{q}^{\prime }$ to AU

*AU*:

07: Receive *r*_*q*_ from *C*_*A*_ and λ_*q*_ from *C*_*B*_

08: $c_{q}=\lambda_{q}^{\prime }-r_{q}$

### 4.5) Example of kNN classification

**Fig 4:**
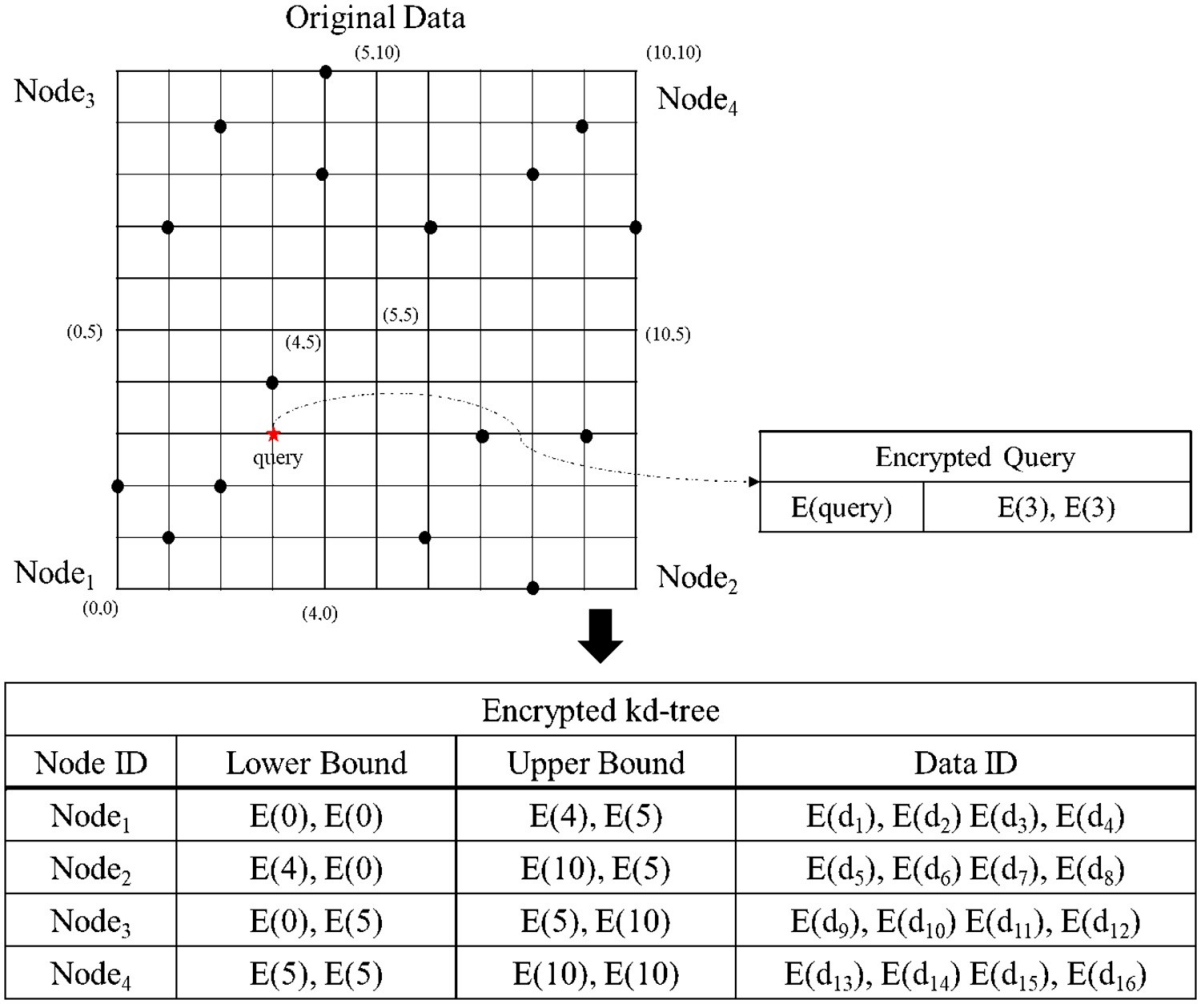
Data and query used in example.

**Fig 5:**
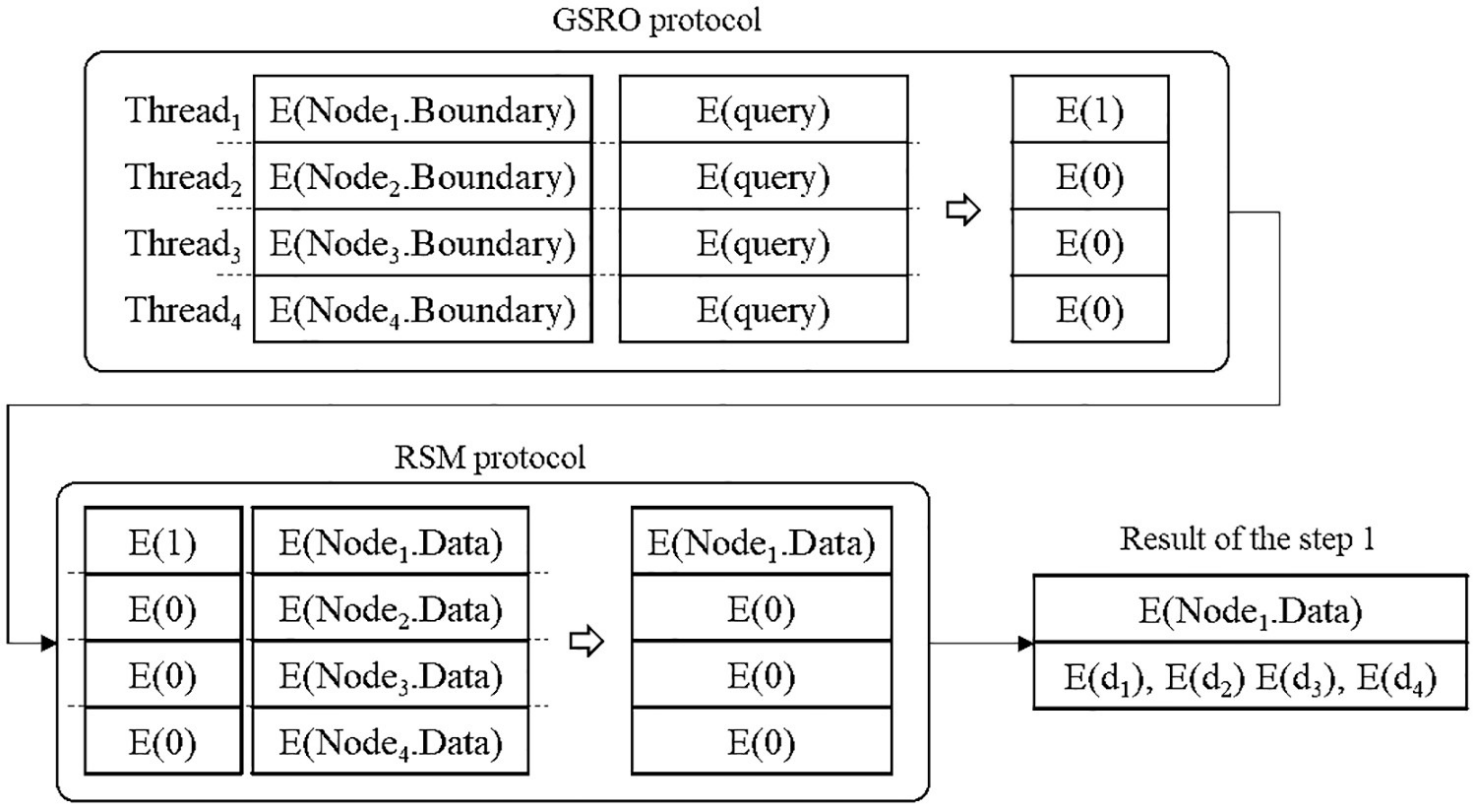
Example of secure index search phase.

**Fig 6:**
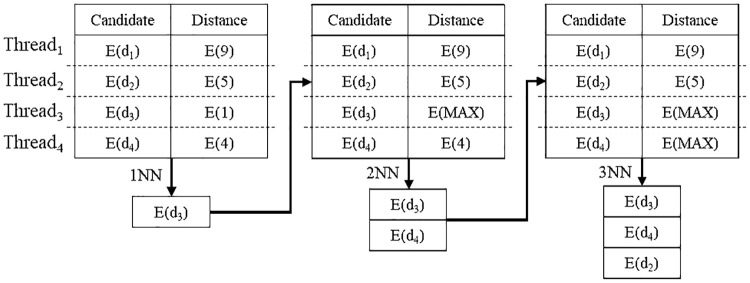
Example of kNN search phase.

**Fig 7:**
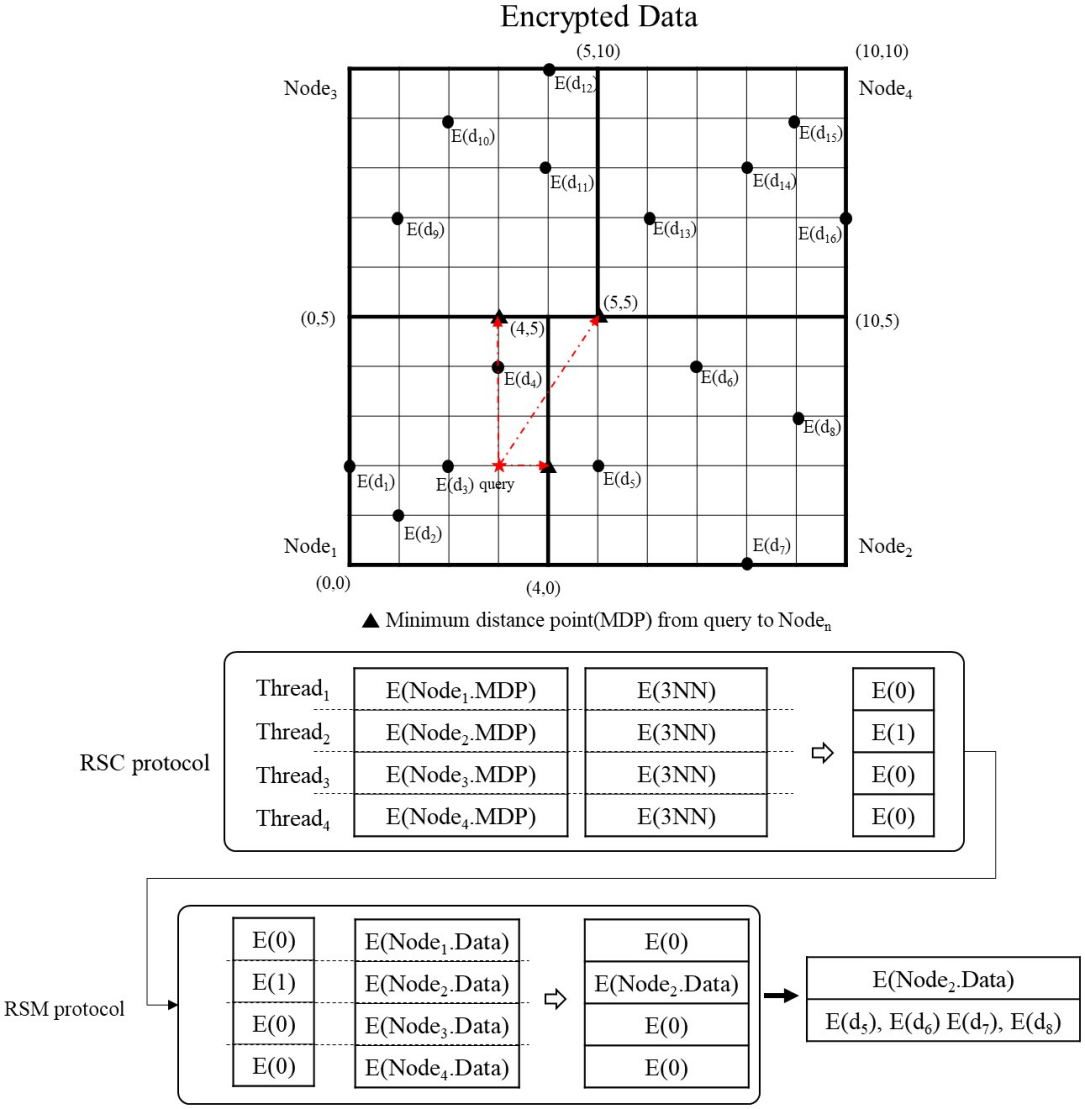
Example of index search in kNN verification phase.

**Fig 8:**
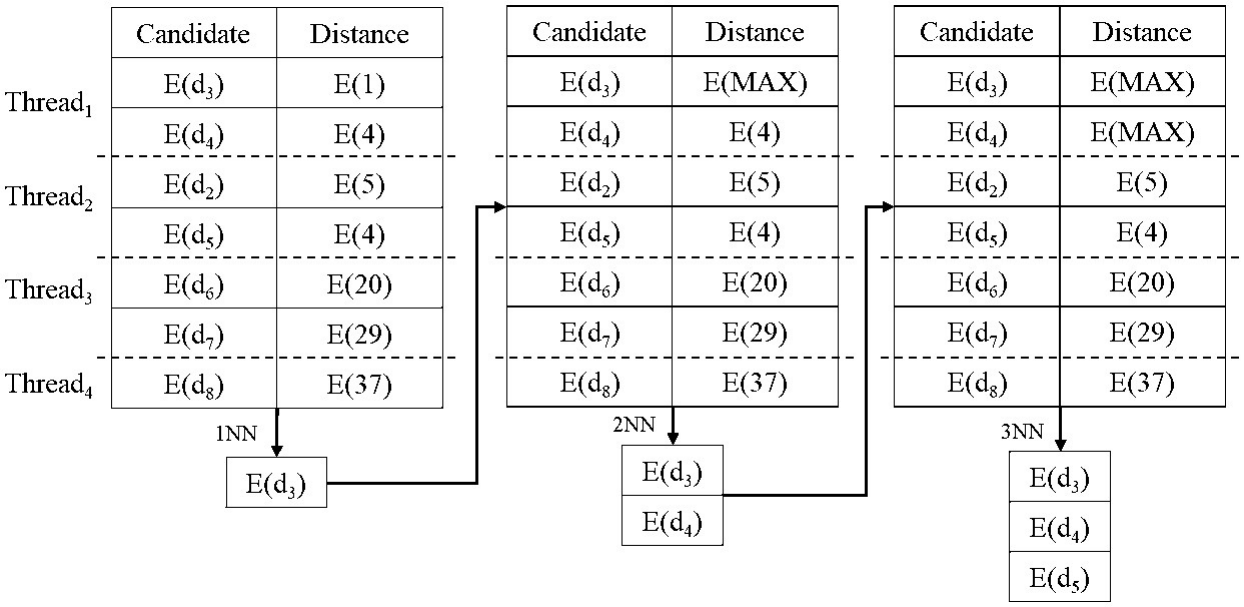
Example of k-nearest neighbor search in kNN verification phase.

**Fig 9:**
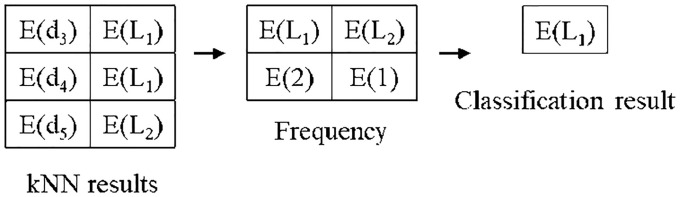
Example of kNN classification phase.

Here, an example of the proposed secure kNN classification algorithm is described. Assume that the original data is indexed and encrypted by using the kd-tree, as shown in Fig 4. The encrypted kd-tree contains 4-fold attributes for each leaf node, i.e., a node identifier (ID), an encrypted lower bound of the node, an encrypted upper bound of the node, and the encrypted data. Fig 5 shows how to extract data in a selected node through the secure index search phase. First, *C*_*A*_ sends a node identifier (ID), an encrypted lower bound, an encrypted upper bound, an encrypted query to all the threads. In each thread, the algorithm performs the GSRO protocol to determine whether a node includes the query or not. If a node includes the query, the GSRO protocol returns *E*(1). Otherwise it returns *E*(0). Second, the algorithm performs the RSM protocol by multiplying the encrypted data in each node(*E*(*node*_*z*_.*data*)) and the results of the GSRO protocol. As a result, *E*(*node*_*z*_.*data*) is returned only if the result of the GSRO is *E*(1). Finally, the algorithm can safely obtain the encrypted data by merging the results of the RSM protocol. Fig 6 shows how to obtain kNN candidates through the k-nearest neighbors search phase. First, the algorithm selects the encrypted data which has the minimum distance from the query by using the GSMIN_*n*_ protocol. In Fig 6, *E*(*d*_3_) is selected as 1NN because the distance of *d*_3_ is the minimum. Second, the algorithm sets the distance of the selected data to the maximum value for excluding the selected data. Therefore, the distance of *E*(*d*_3_) is set to *E*(MAX). Finally, the algorithm is repeated until the *k*^*th*^ nearest data is selected. In the same way, *E*(*d*_4_) and *E*(*d*_2_) are selected as 2NN and 3NN, respectively. As a result, the algorithm can safely select the k number of nearest neighbors. Figs 7 and 8 show the examples of index search and k-nearest neighbor search in the kNN verification phase, respectively. In each thread, the algorithm calculates the shortest distance *E*(*spdist*_*z*_) between the query and a leaf node(*node*_*z*_), and compares *E*(*spdist*_*z*_) with *E*(*dist*_*k*_). If *E*(*spdist*_*z*_) is smaller than *E*(*dist*_*k*_), the data in the *node*_*z*_ is extracted. In Fig 7, because *E*(*spdist*_2_), i.e., (*E*(1)), is smaller than *E*(*dist*_*k*_), i.e., (*E*(5)), *node*_2_ is selected. Fig 8 shows how to obtain the final kNN. The algorithm merges the kNN candidates and obtains the final k-nearest neighbors. In the kNN classification phase, the algorithm calculates the frequency of labels in *E*(*L*′). Because the frequency of *E*(*L*_1_) is the highest in kNN, *E*(*L*_1_) is selected as the final result, as shown in Fig 9.

## 5 Random value pool’s security proof

### 5.1) Security proof of the secure protocols

In this section, we describe the security proof of the SME and the GSCE protocols proposed in Section 3. To prove that the proposed protocols are secure under the semi-honest model, we show that the simulated images of the proposed protocols are computationally indistinguishable from their actual execution images. Security proof of the SME protocol: We describe the security proof of the SME protocol by analyzing the security of the execution images of *C*_*A*_ and *C*_*B*_. First, the execution image on *C*_*B*_ side, i.e., $\prod_{C_{B}}\left(SME\right)$, is shown in Eq 8. Here, $E(v_{1}^{\prime })$ and $E(v_{2}^{\prime })$ are the encrypted data received from *C*_*A*_ (line 1∼2 of Algorithm 1), $v_{1}^{\prime }$ and $v_{2}^{\prime }$ and are obtained through the decryption of $E(v_{1}^{\prime })$ and $E(v_{2}^{\prime })$, respectively. Also, *α* is a result which is calculated by the SME protocol using $v_{1}^{\prime }$ and $v_{2}^{\prime }$ on *C*_*B*_ side.

$$\begin{array}{c} \prod_{C_{B}}\left(SME\right)=\left\{<E\left(v_{1}^{\prime }\right),E\left(v_{2}^{\prime }\right),v_{1}^{\prime },v_{2}^{\prime }>,\alpha \right\} \end{array}$$ (8)

For example, assume that $\prod C_{B^{s}}\left(SME\right)=\left\{<E\left(s_{1}^{\prime }\right),E\left(s_{2}^{\prime }\right),s_{1}^{\prime },s_{2}^{\prime }>,s_{3}\right\}$ is the simulated execution image using the SME protocol on *C*_*B*_ side. Here, $E(s_{1}^{\prime })$ and $E(s_{2}^{\prime })$ are the non-deterministic numbers selected in *Z*_*N*^2^_, and $s_{1}^{\prime }$ and $s_{2}^{\prime }$ are the indistinguishable numbers which are added by each value in the random value pool. $s_{3}^{\prime }$ is the result of the SME protocol using $s_{1}^{\prime }$ and $s_{2}^{\prime }$ on *C*_*B*_ side. Because the SME protocol is implemented based on the Paillier cryptosystem, it can support semantic security. Therefore, $E(s_{1}^{\prime })$ and $E(s_{2}^{\prime })$ are computationally indistinguishable from $s_{1}^{\prime }$ and $s_{2}^{\prime }$, respectively. $s_{3}^{\prime }$ is indistinguishable from $s_{1}^{\prime }$ and $s_{2}^{\prime }$ because $s_{3}^{\prime }$ is calculated by multiplying two indistinguishable numbers in *C*_*A*_, $s_{1}^{\prime }$ and $s_{2}^{\prime }$. Therefore, it can be said that $\prod_{C_{B}}\left(SME\right)$ is computationally indistinguishable from $\prod_{C_{B^{S}}}\left(SME\right)$. Because *C*_*B*_ can check only the result (e.g., *α*) of the multiplication between the non-deterministic numbers (e.g., $v_{1}^{\prime }$ and $v_{2}^{\prime }$), *C*_*B*_ cannot obtain the original data while performing the SME protocol. Meanwhile, the execution image of *C*_*A*_ is $\prod_{C_{A}}\left(SME\right)=\{E(\alpha )\}$ such that *E*(*α*) from *C*_*B*_ can be regarded as the result of the SME protocol. Suppose that the simulated image of *C*_*A*_ is $\prod_{C_{A^{S}}}\left(SME\right)=\{E(s_{4})\}$, where *E*(*s*_4_) is randomly generated from *Z*_*N*^2^_. Therefore, *E*(*α*) is computationally indistinguishable from *E*(*s*_4_). According to the above analyses, there is no information leakage both at *C*_*A*_ and *C*_*B*_ side. Therefore, we can conclude that the proposed SME protocol is secure under the semi-honest adversarial model. Security proof of the GSCE protocol: We describe the security proof of the GSCE protocol by analyzing the security of the execution images of *C*_*A*_ side and *C*_*B*_ side. First, the execution image on *C*_*B*_ side, i.e., $\prod_{C_{B}}\left(GSCE\right)$, is shown in Eq 9. Here, $E(\sigma_{1}^{\prime })$ and $E(\sigma_{2}^{\prime })$ refer to the encrypted data received from *C*_*A*_ (line 1∼2 of Algorithm 2), and both $\sigma_{1}^{\prime }$ and $\sigma_{2}^{\prime }$ are obtained through decryption of $\sigma_{1}^{\prime }$ and $\sigma_{2}^{\prime }$, respectively. Also, *β* is the result which is calculated by the GSCE protocol using $\sigma_{1}^{\prime }$ and $\sigma_{2}^{\prime }$ on *C*_*B*_ side.

$$\begin{array}{c} \prod_{C_{B}}\left(GSCE\right)=\left\{<E\left(\sigma_{1}^{\prime }\right),E\left(\sigma_{2}^{\prime }\right),\sigma_{1}^{\prime },\sigma_{2}^{\prime }>,\beta \right\} \end{array}$$ (9)

For example, assume that $C_{B^{S}}\left(GSCE\right)=\left\{<E\left(s_{1}^{\prime }\right),E\left(s_{2}^{\prime }\right),s_{1}^{\prime },s_{2}^{\prime }>,s_{3}\right\}$ for the simulated execution image using the GSCE protocol on *C*_*B*_ side. Here, $E(s_{1}^{\prime })$ and $E(s_{2}^{\prime })$ are the non-deterministic numbers selected in *Z*_*N*^2^_, and both $s_{1}^{\prime }$ and $s_{2}^{\prime }$ are the indistinguishable numbers selected in the random value pool. $s_{3}^{\prime }$ is the result of the GSCE protocol using $s_{1}^{\prime }$ and $s_{2}^{\prime }$ on *C*_*B*_ side. Because the GSCE protocol is implemented based on the Paillier cryptosystem, it can support semantic security. Therefore, $E(s_{1}^{\prime })$ and $E(s_{2}^{\prime })$ are computationally indistinguishable from $s_{1}^{\prime }$ and $s_{2}^{\prime }$, respectively. $s_{3}^{\prime }$ is indistinguishable from $s_{1}^{\prime }$ and $s_{2}^{\prime }$ because $s_{3}^{\prime }$ is calculated by comparing two indistinguishable numbers in *C*_*A*_, $s_{1}^{\prime }$ and $s_{2}^{\prime }$. Therefore, it can be said that $\prod_{C_{B}}\left(GSCE\right)$ is computationally indistinguishable from $\prod_{C_{B^{S}}}\left(GSCE\right)$. Because *C*_*B*_ can check only the result (e.g., *β*) of the comparison between the non-deterministic numbers (e.g., $\sigma_{1}^{\prime }$ and $\sigma_{2}^{\prime }$), *C*_*B*_ cannot obtain the original data while performing the GSCE protocol. Meanwhile, the execution image of *C*_*A*_ is $\prod_{C_{A}}\left(GSCE)=E(\beta \right)$ such that *E*(*β*) from *C*_*B*_ can be regarded as the result of the GSCE protocol. Suppose that the simulated image of *C*_*A*_ is $\prod_{C_{A}^{S}}\left(GSCE\right)=E(s_{4})$, where *E*(*s*_4_) is randomly generated from *Z*_*N*^2^_. Therefore, *E*(*β*) is computationally indistinguishable from *E*(*s*_4_). According to the above analyses, there is no information leakage both at *C*_*A*_ and *C*_*B*_ side. Therefore, we can conclude that the proposed GSCE protocol is secure under the semi-honest adversarial model

### 5.2) Security proof of the proposed kNN classification algorithm

We prove that the proposed kNN classification algorithm on the encrypted database is safe under the semi-honest attack model. The proposed kNN classification algorithm in the cryptographic database consists of a secure index search phase (Algorithm 3), a kNN search phase (Algorithm 4), a kNN verification phase (Algorithm 5), and a kNN classification phase (Algorithm 6). To show that the proposed secure kNN classification algorithm is safe under the semi-honest attack model, security analysis is performed at each execution phase. First, because the secure index search phase is composed of the GSRO protocol [17] which has been proven to be safe, the Algorithm 3 is safe under the semi-honest attack model by composition theory [17]. Second, the kNN search phase is safe in *C*_*A*_ side, because *C*_*A*_ performs the ESSED, *SMIN*_*n*_ and SM protocols which have been proven to be safe in the previous studies [16, 17]. Even though the kNN search phase decrypts the received data from *C*_*A*_, *C*_*B*_ cannot extract the original data. This is because the data received from *C*_*A*_ is modified by raising the original data to the power of a random integer and applying a shuffling function. Therefore, according to the composition theory, Algorithm 4 is safe under the semi-honest attack model. Third, the images which are generated by the kNN verification phase are the same as those generated by Algorithms 3 and 4. Therefore the kNN verification phase (Algorithm 5) is safe under the semi-honest attack model. Lastly, the kNN classification phase (Algorithm 6) is safe under the semi-honest attack model because Algorithm 6 has been proven safe in the previous work [16, 17]. As a result, all the phases of the proposed secure kNN classification algorithm is safe under the semi-honest attack model.

## 6 Performance analysis

Because there is no privacy-preserving parallel kNN Classification algorithm, we compare our privacy-preserving parallel kNN classification algorithm with the extension of existing works. That is, we make parallel SkNNC-M by extending B. K. Samanthula et. al.’s work [16] in a naive way so that it may operate in a multi-core environment. We make parallel SkNNC-G by extending H. J. KIM et. al.’s work [17] in the same way. For performance evaluation, three algorithms were implemented by using C++ under an Intel(R) Xeon(R) CPU E5–2630 v4 @ 2.20GHz and 64GB (16GB × 4AE) DDR3 UDIMM 1600MHz in a Linux Ubuntu 18.04.2 environment. We compare three parallel algorithms in terms of the query processing time by varying the number of data, the number of k, the level of the kd-tree, the number of the data dimension, and the number of threads. We use both a synthetic dataset and real dataset [28] for our experiments.

### 6.1) Performance analysis of kNN classification algorithm for synthetic dataset

Table 4 shows the parameters used in the performance evaluation for the synthetic dataset. For the synthetic dataset, we randomly generate 30,000 integer data with 12 dimensions. The domain of data is ranged from 0 to 212. We do an experiment to find the optimal value of the level of kd-tree(h). It is shown that the performances of both SkNNC-G and the proposed algorithm are best when h is 7. So, we set h to 7 in our experiment.

**Table 4:** Parameters used in performance evaluation for synthetic data.

Parameter	Values	default
the number of data(n)	5k,10k,20k,30k	10k
*k*	5, 10, 15, 20	10
level of kd-tree(h)	5, 6, 7, 8, 9	7
the number of threads	1, 2, 5, 10	10
the number of data dimension(m)	3, 6, 9, 12	6
Size of encryption key(K)	512	-
bit size for data domain	12	-

**Fig 10:**
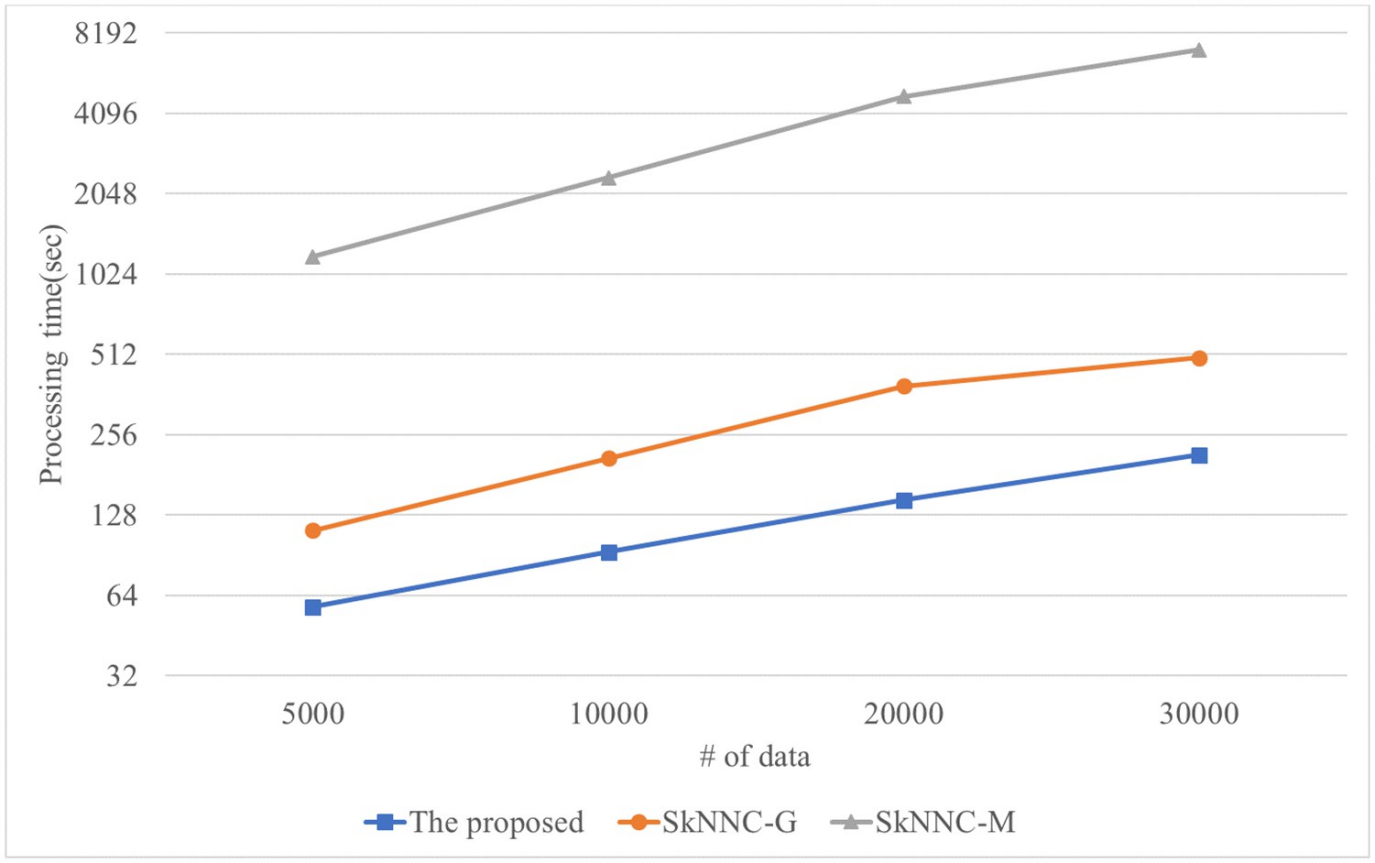
Processing time with varying the number of data.

**Fig 11:**
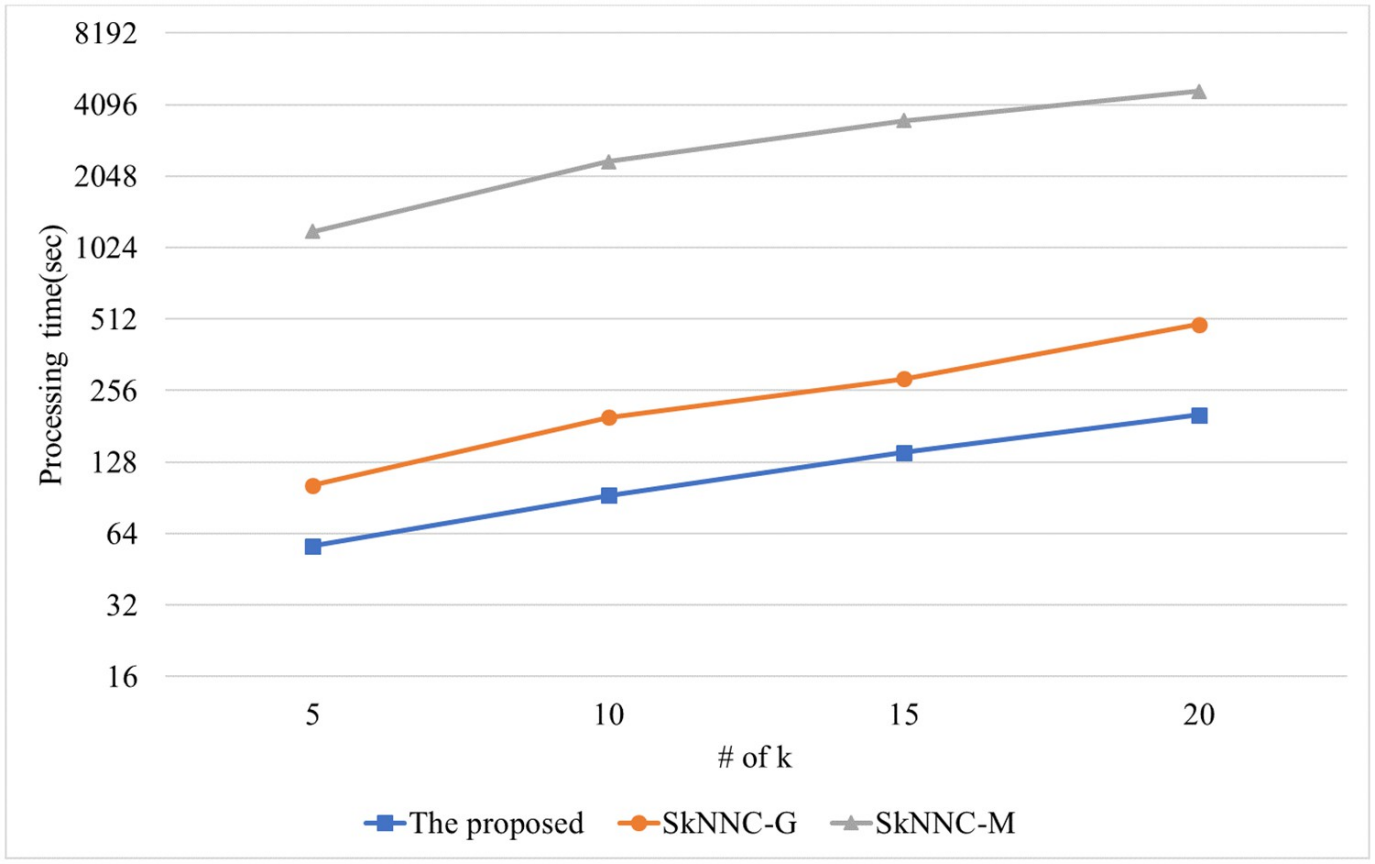
Processing time with varying k.

The performance of the kNN classification algorithms is evaluated for synthetic data. Fig 10 shows the performance of the proposed algorithm, parallel SkNNC-M, and parallel SkNNC-G according to the number of data. When n = 30k, the proposed algorithm, parallel SkNNC-G, and parallel SkNNC-M require 215, 497, and 7,089 seconds, respectively. That is, the proposed algorithm shows 2.3 times better performance than parallel SkNNC-G and 32 times better performance than parallel SkNNC-M. This is because our secure protocols (SME and GSCE protocols) can reduce the number of data encryptions by selecting an encrypted value from the random value pool instead of generating it, as mentioned in Table 3. Fig 11 shows the performance of the proposed algorithm, parallel SkNNC-M, and parallel SkNNC-G according to k. When k = 20, the proposed parallel algorithm, parallel SkNNC-G, and parallel SkNNC-M require 202, 487, and 4,658 seconds, respectively. That is, the proposed algorithm shows 2.4 times better performance than parallel SkNNC-G and 23 times better performance than parallel SkNNC-M. The reason is the same as mentioned in Fig 10.

**Fig 12:**
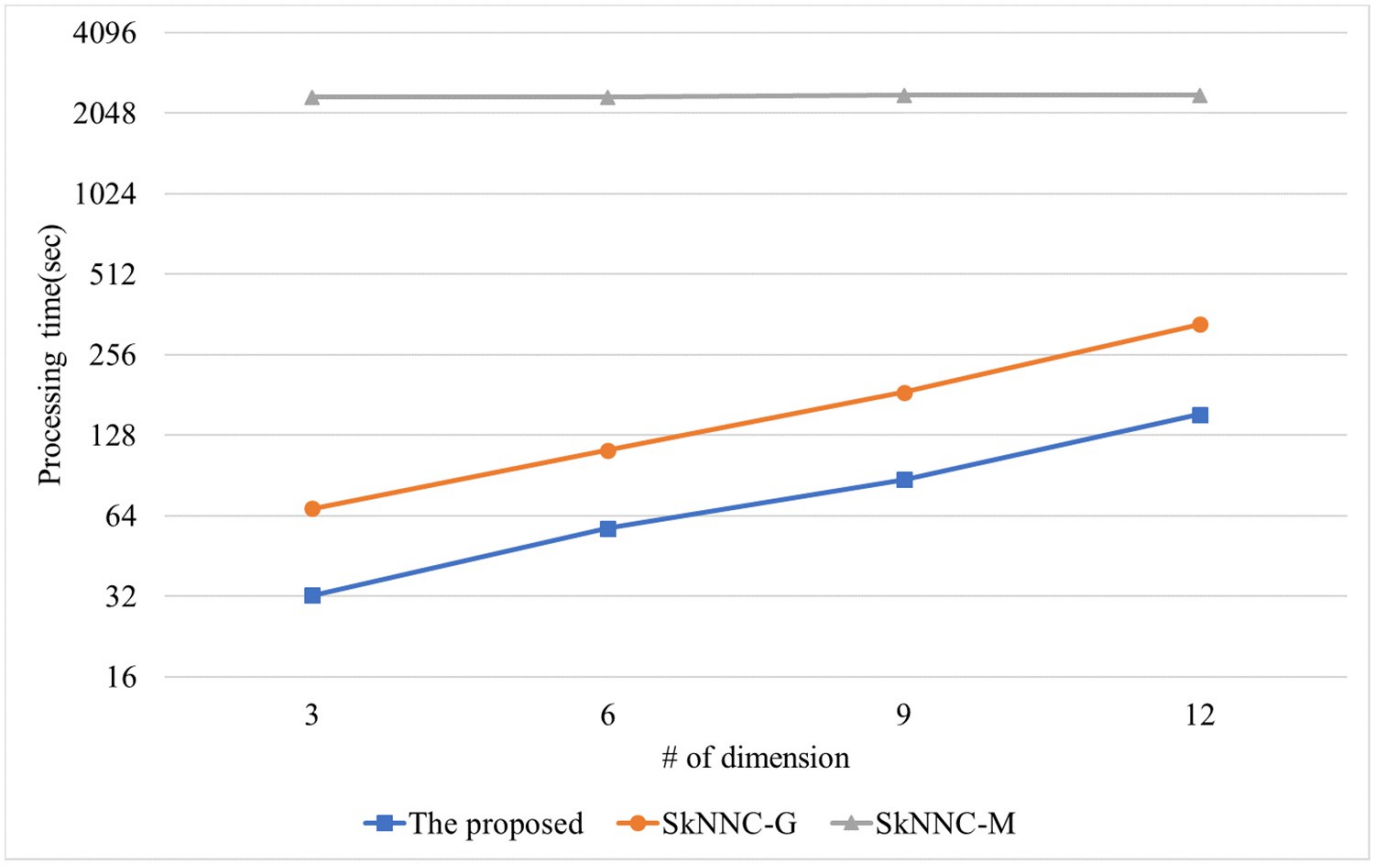
Processing time with varying the number of dimensions.

**Fig 13:**
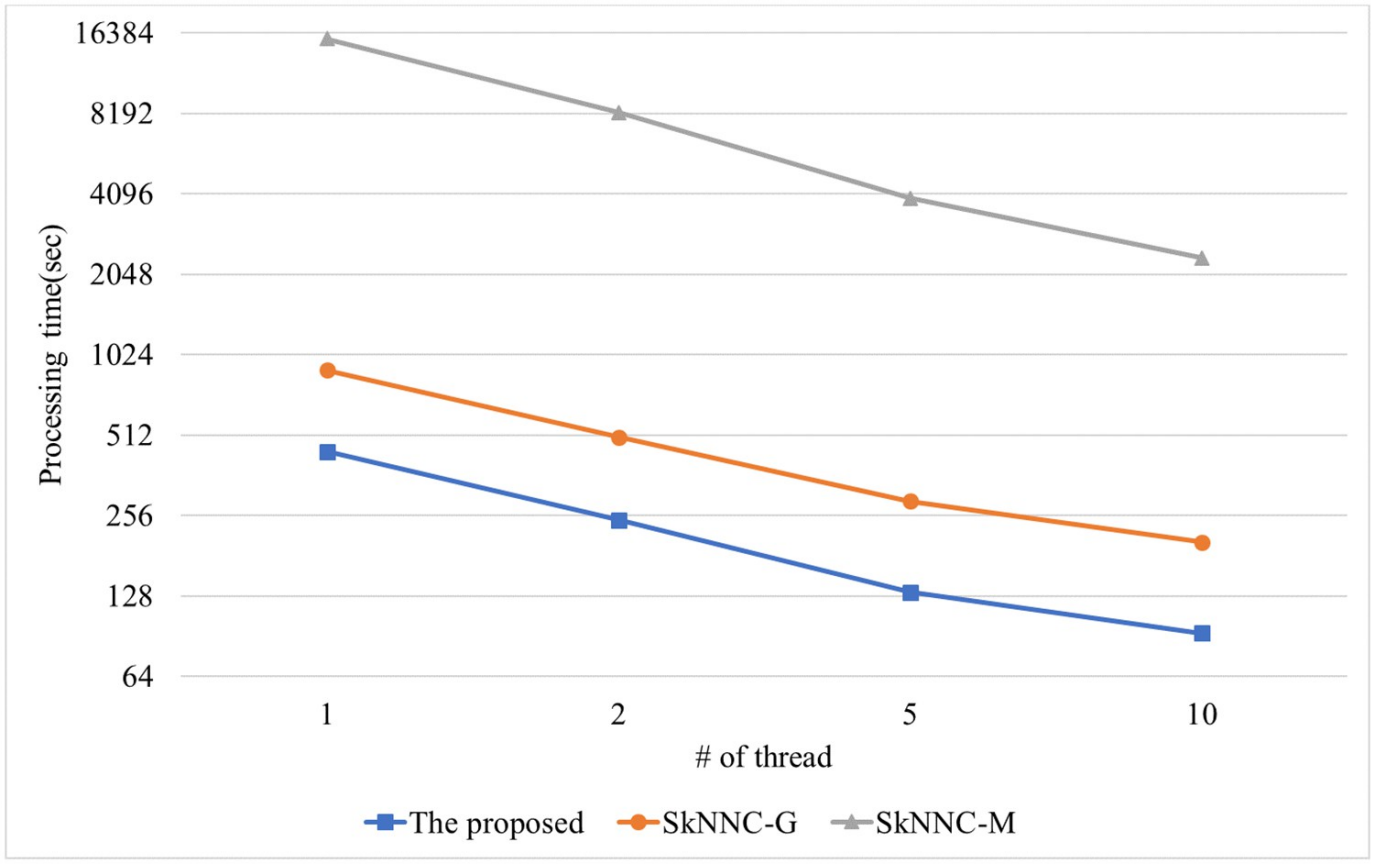
Processing time with varying the number of threads.

Fig 12 shows the performance of the proposed algorithm, parallel SkNNC-M, and parallel SkNNC-G according to the number of data dimension(m). When m = 6, the proposed parallel algorithm, parallel SkNNC-G, and parallel SkNNC-M require 57, 112, and 2,353 seconds, respectively. That is, the proposed algorithm shows 2 times better performance than parallel SkNNC-G and 15 times better performance than parallel SkNNC-M. The reason is the same as mentioned in Fig 10. Fig 13 shows the performance of the proposed algorithm, parallel SkNNC-M, and parallel SkNNC-G according to the number of threads. When the number of threads = 1(single-core), the proposed algorithm, parallel SkNNC-G, and parallel SkNNC-M require 443, 894, and 15,572 seconds, respectively. That is, the proposed algorithm shows 2 times better performance than parallel SkNNC-G and 35 times better performance than parallel SkNNC-M. This is because our secure protocols (SME and GSCE protocols) can reduce the number of data encryptions by selecting an encrypted value from the random value pool instead of generating it. When the number of threads = 10, the proposed algorithm, parallel SkNNC-G, and parallel SkNNC-M require 93, 203, and 2350 seconds, respectively. That is, the proposed algorithm shows 2.1 times better performance than parallel SkNNC-G and 25 times better performance than parallel SkNNC-M. Because a thread performs secure protocols concurrently without interfering with each other, query processing time linearly decreases as the number of threads increases. As a result, our parallel algorithm shows better performance than the existing algorithms in a multi-core environment.

### 6.2) Performance analysis of kNN classification algorithm for real dataset

Table 5 shows the parameters used in the performance evaluation for real data. For this, we used a chess dataset [28] generated by a chess endgame database for white king and rook against black king. The chess dataset aims to classify the optimal depth of win for white. With the real dataset, we do an experiment to find the optimal value of the level of kd-tree(h). It is shown that the performances of both SkNNC-G and the proposed algorithm are best when h is 7. So, we set h to 7 in our experiment.

**Table 5:** Parameters used in performance evaluation for real data.

Parameter	Values	default
the number of data(n)	28056	-
*k*	5, 10, 15, 20	10
level of kd-tree(h)	7	-
the number of threads	1, 2, 5, 10	10
the number of data dimension(m)	6	-
Size of encryption key(K)	512	-
bit size for data domain	12	-

**Fig 14:**
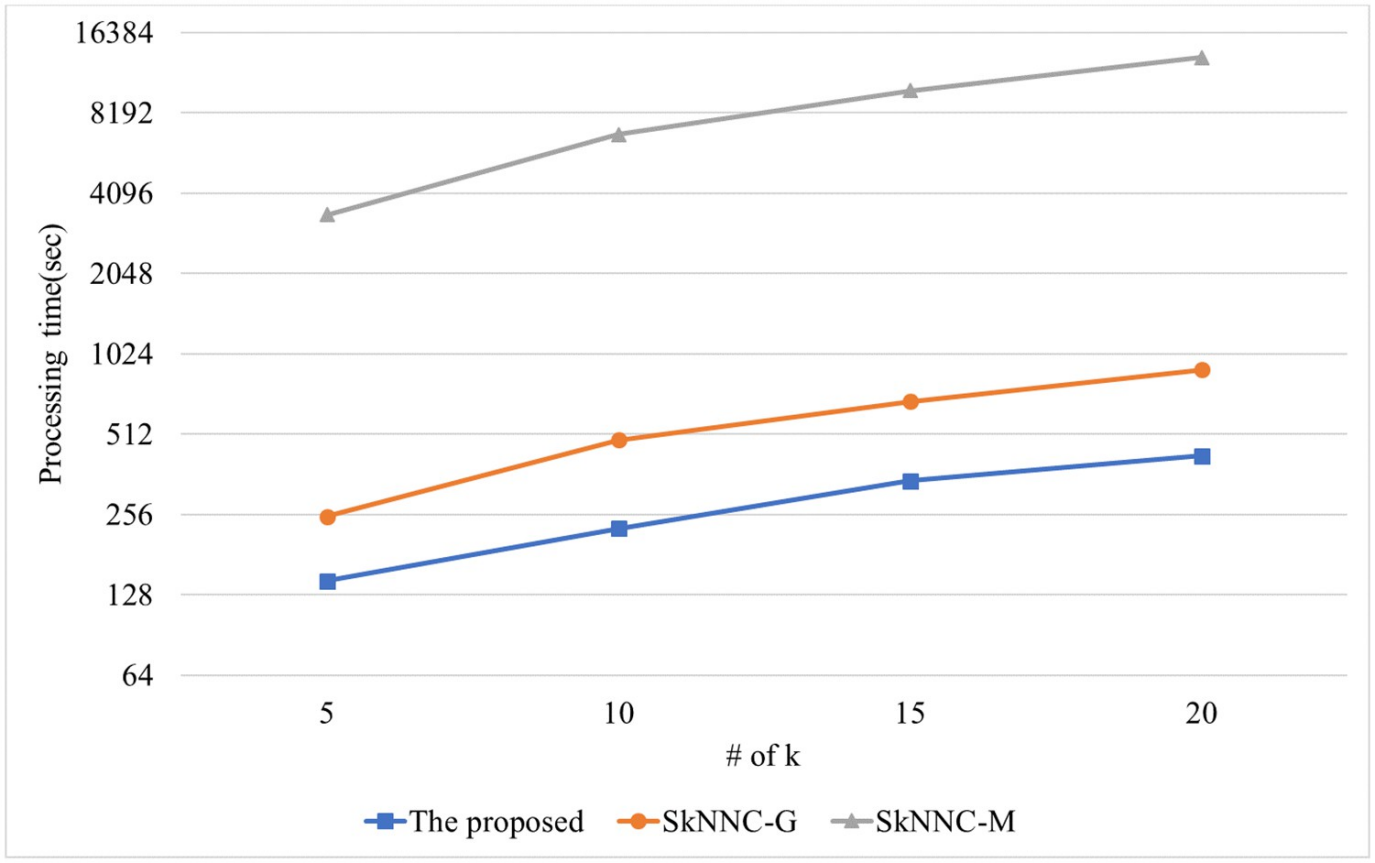
Processing time with varying k.

**Fig 15:**
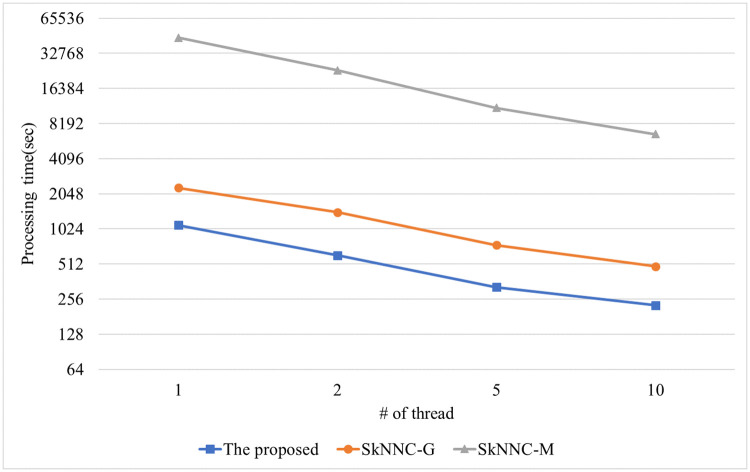
Processing time with varying the number of threads.

Fig 14 shows the performance of the proposed algorithm, parallel SkNNC-M, and parallel SkNNC-G according to k. When k = 20, the proposed algorithm, parallel SkNNC-G, and parallel SkNNC-M require 425, 894, and 13,175 seconds, respectively. That is, the proposed algorithm shows 2 times better performance than parallel SkNNC-G and 27 times better performance than parallel SkNNC-M. This is because our algorithm uses both SME and GSCE protocols which can reduce the number of data encryptions by selecting an encrypted value from the random value pool. Fig 15 shows the performance of the proposed algorithm, parallel SkNNC-M, and parallel SkNNC-G according to the number of threads. When the number of threads = 1 (single-core), the proposed algorithm, parallel SkNNC-G, and parallel SkNNC-M require 1106, 2306, and 44,570 seconds, respectively. That is, the proposed algorithm shows 2 times better performance than parallel SkNNC-G and 40 times better performance than parallel SkNNC-M. The reason is the same as mentioned in Fig 14. When the number of threads = 10, the proposed algorithm, parallel SkNNC-G, and parallel SkNNC-M require 227, 487, and 6,639 seconds, respectively. That is, the proposed algorithm shows 2 times better performance than parallel SkNNC-G and 29 times better performance than parallel SkNNC-M. Because a thread performs secure protocols concurrently without any interference of each other, it can be seen that query processing time linearly decreases as the number of threads increases.

### 6.3) Theoretical analysis of the proposed algorithm in terms of privacy

Assuming that an attacker does not have any information of original data items, an adversary needs tremendous time to obtain the original plaintext from paillier cryptosystem while using a brute force attack. It means that it is impossible to do an experiment to prove data protection, query protection and access pattern protection. Therefore, instead of experimental analysis, we conduct the theoretical analysis of data privacy, query privacy and access pattern privacy to support the security analysis of the proposed algorithm. For this, we estimate the time complexity it takes for the original data to be exposed and calculate the probability of access pattern leakage.

#### 6.3.1 Theoretical analysis of data privacy

In *C*_*A*_, an attacker only obtains the ciphertext of data. Because the data is protected by the paillier cryptosystem, the security performance is measured through the time complexity of the brute force attack to break down the paillier cryptosystem. Our paillier cryptosystem uses 512-bit encryption key size. Assuming that CPU cycle is 4GHz, the time required to decrypt the ciphertext by changing the key is as shown in Eq (10).

$$\begin{array}{c} BFAtime\left(sec\right)=\frac{2^{512}}{4GHz}\approx \frac{1.3\times 10^{154}}{4GHz} \end{array}$$ (10)

**Fig 16:**
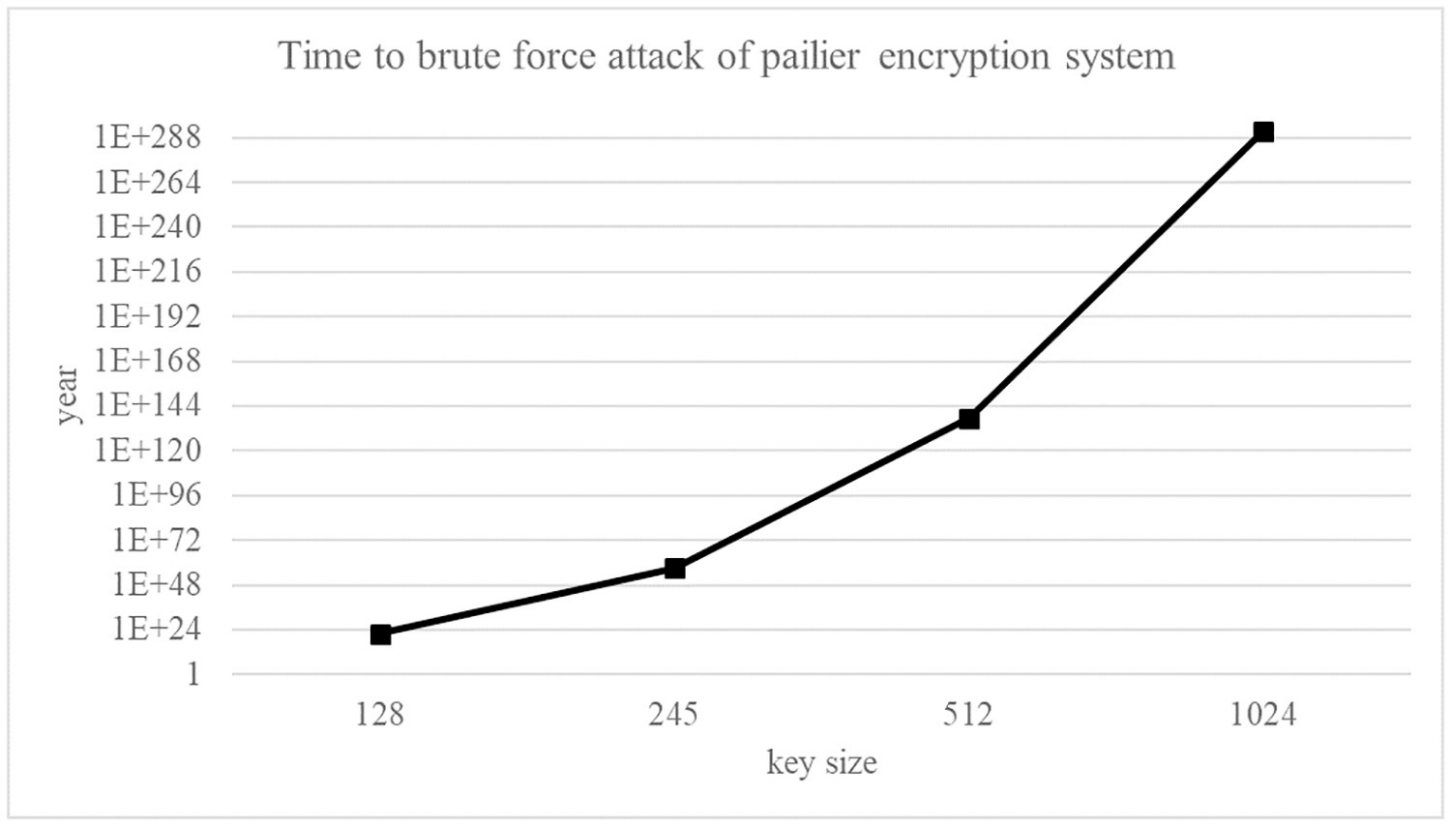
Brute force attack time with varying the key size.

It is impossible to break down a paillier cryptosystem because it takes about 4.2 × 10^146^ years with 512-bit key size. It means that the proposed privacy preserving kNN classification algorithm is secure in terms of data privacy even if the ciphertext is exposed. Fig 16 shows the time taken for a brute force attack in *C*_*A*_ as the key size is changed. In *C*_*B*_, an attacker only obtains a plaintext data which adds a random number to the original data. In the paillier cryptosystem, because the range of the plaintext data is 0 ≤ *m* ≤ 2^512^, brute force attack time in *C*_*B*_ has the same as that in *C*_*A*_.

#### 6.3.2 Theoretical analysis of query privacy

In *C*_*A*_, an attacker only obtains the ciphertext of query. Because the query is protected by the paillier cryptosystem, the security performance is measured through the time complexity of the brute force attack to break down the paillier cryptosystem. Since our paillier cryptosystem uses 512-bit encryption key size, the time required to decrypt the ciphertext by changing the key is as shown in 10, where CPU cycle is 4GHz. It is impossible to break down a paillier cryptosystem because it takes about 4.2 × 10^146^ years with 512-bit key size. It means that the proposed privacy preserving kNN classification algorithm is secure in terms of query privacy even if the ciphertext is exposed. The times taken for a brute force attack in *C*_*A*_ is the same as that of data privacy in *C*_*A*_ (Fig 16). In *C*_*B*_, query privacy is preserved because *C*_*B*_ does not receive the query.

#### 6.3.3 Theoretical analysis of access pattern privacy

The access pattern means the sequence of accessing a data item. In the proposed algorithm, the sequence of accessing a data item consists of the leaf node access of kd-tree and data access in the leaf node. In *C*_*A*_, an attacker only obtains the ciphertext of leaf node. Because all the leaf nodes have the same number of data items, an attacker cannot distinguish the leaf node by using density of data items. If the kd-tree level is *h*, the number of leaf node is 2^*h*−1^. The probability that an attacker can distinguish a node(*node*_*i*_) from the others, i.e., *P*(*node*_*i*_), is $\frac{1}{2^{h-1}}$. Because *node*_*i*_ includes the same number of data items as fanout, the probability that an attacker can distinguish a data item from the others in *node*_*i*_, i.e., *P*(*node*_*i*_.*data*_*j*_), is $\frac{1}{fanout}=\frac{1}{\frac{the\;number\;of\;data}{2^{h-1}}}=\frac{2^{h-1}}{n}$. Therefore, the probability of data access pattern leakage (*P*_*APL*_) is shown in Eq (11).

$$\begin{array}{c} P_{APL}=P({node}_{i})\times P({node}_{i}.{data}_{j})=\frac{1}{2^{h-1}}\times \frac{2^{h-1}}{n}=\frac{1}{n} \end{array}$$ (11)

*P*_*APL*_ is equal to the probability that an attacker distinguishes a specific data item from the others in the entire data items. Therefore, the proposed algorithm can preserve the access pattern privacy in *C*_*A*_. In *C*_*B*_, access pattern privacy is preserved because *C*_*B*_ does not have any data item.

## 7 Discussion

### 7.1 Impact of hiding data access patterns

The data access pattern is one of the most important factors for privacy preservation. If an attacker possesses the order or the frequency of data, he/she can infer the original data by using data access patterns. Therefore, hiding data access patterns is as important as encrypting data. First, B. Yao et al.’s work [21] proposed a secure kNN classification algorithm using the Voronoi diagram [22]. However, the order of accessing the Voronoi diagram is distinguishable and an attacker can partially infer the original data from the query. Second, J. Du and F. Bian’s work [25] proposed a kNN classification algorithm using an order-preserving index. However, the index access patterns are exposed because the order of accessing the index can be easily obtained from the query. This allows an attacker to easily infer the original data if he/she has an index access pattern. Meanwhile, our algorithm uses the Paillier cryptosystem which supports semantic security for data protection. As a result, all of the ciphertext is indistinguishable and secure from frequency-based attacks. In addition, the kd-tree filtering technique used in our algorithm is secure from the exposure of data access patterns because our algorithm accesses only the encrypted leaf nodes of the kd-tree without accessing the index by using a top-down approach. Therefore, our algorithm can hide the data access patterns.

### 7.2 Impact of parallel algorithm with garbled circuit

First, a garbled circuit is used for efficient processing of secure protocols. B. K. Samanthual et al.’s work [16] has high overhead by using a secure protocol based on the comparison of binary array. To overcome this problem, our secure protocols use a garbled circuit that performs a fast and secure comparison operation in the state of the ciphertext. Second, the existing algorithms do not use parallelism for the privacy-preserving classification algorithm [16, 17, 25]. On the contrary, our algorithm proposes a parallel classification algorithm adopting the garbled circuit. Our algorithm performs three phases in parallel: index searching, kNN searching and kNN verification. As shown in our performance evaluation, our parallel classification algorithm shows performance improvement in proportion to the number of threads.

### 7.3 Impact of encrypted random value pool

In our secure system, we use two-party computation for the parallel kNN classification algorithm. Thus, we need to prevent *C*_*B*_ from extracting meaningful information while executing secure protocols. For this, *C*_*A*_ generates a random value r from *Z*_*N*_ and encrypts *r* by using the Paillier cryptosystem. Then, *C*_*A*_ adds the encrypted random value *E*(*r*) to the encrypted plaintext *E*(*m*) by computing *E*(*m* + *r*) = *E*(*m*) × *E*(*r*). Because *m*±*r* is independent from *m*, *C*_*B*_ cannot obtain meaningful information with decryption. However, adding a random value to the ciphertext in the Paillier cryptosystem leads to performance degradation because both encryption and decryption operations require higher computation cost than other encrypted operations. In the Secure Multiplication protocol, both B. K. Samanthula et al.’s work and H. Kim et al.’s work require three times of the encryption: 2 encryptions for random values at *C*_*A*_ and 1 encryption for the result of multiplication at *C*_*B*_. Meanwhile, our algorithm requires only one encryption for the result of multiplication at *C*_*B*_ because it selects the encrypted random values from the random value pool without encrypting the random values at *C*_*A*_. In the Secure Compare protocol, B. K. Samanthula et al.’s work requires *log*_2_ *D* times of encryption where *D* is a data domain. H. Kim et al.’s work requires three times of the encryption: 2 encryptions for random values at *C*_*A*_ and 1 encryption for the result of the comparison between two values at *C*_*B*_. Meanwhile, our algorithm requires only one encryption for the result of comparison at *C*_*B*_ by using the random value pool. Therefore, our algorithm can reduce the amount of computation cost for encryption by using the encrypted random value pool.

### 7.4 Practical example of proposed kNN classification

The proposed secure kNN classification algorithm can be used in various fields. For example, first, it can be used to diagnose a disease by classifying the patterns of the patient’s symptoms [29]. Because the existing disease diagnosis system depends on only the doctor’s knowledge and experience, it may cause damage to patients due to misdiagnosis. Therefore, kNN classification algorithms can help doctors classify the pattern of the patient’s symptoms so as to diagnose what kind of disease it is. However, because patients’ information contains sensitive data, such as past medical history, family history and allergies, the proposed privacy-preserving kNN classification algorithm can be used to protect the sensitive data of patients. Second, the proposed privacy-preserving kNN classification algorithm can be used to solve the problem of insurance coverage recommendation where insurance companies provide the most suitable coverage for customers [30]. The insurance coverage recommendation classifies customers’ grades based on various customers’ information, such as movement patterns and lifestyles. To perform the classification of customers’ grades, the proposed privacy-preserving kNN classification algorithm can be used to protect the personal information of customers.

## 8 Conclusion

In this paper, we proposed a parallel kNN classification algorithm over encrypted data to preserve data privacy, query privacy, and access pattern privacy in cloud computing. To reduce the computation cost for encryption, we proposed two secure protocols, SME and GSCE, which support secure multi-party computation by using an encrypted random value pool. To reduce the query processing time, we not only designed a parallel algorithm, but also adopted a garbled circuit. In addition, we proved that our algorithm over the encrypted database is safe under the semi-honest attack model. Through our performance evaluation, our algorithm showed about 2∼25 times better performance compared with the existing algorithms. For future work, we plan to apply our parallel query processing algorithm to secure k-Means clustering.

## Supporting information

S1 File (BST)
