# Correction: Privacy-preserving parallel kNN classification algorithm using index-based filtering in cloud computing

**DOI:** 10.1371/journal.pone.0274981

**Published:** 2022-09-15

**Authors:** Yong-Ki Kim, Hyeong-Jin Kim, Hyunjo Lee, Jae-Woo Chang

There are errors in the Funding statement. The correct Funding statement is as follows: This work was supported by a National Research Foundation of Korea (NRF) grant funded by the Korean government (MSIT) (No.2019R1I1A3A01058375). This paper was also supported by research funds of Jeonbuk National University in 2020. The funders had no role in study design, data collection and analysis, decision to publish, or preparation of the manuscript.
